# Deciphering a global source of non-genetic heterogeneity in cancer cells

**DOI:** 10.1093/nar/gkad666

**Published:** 2023-08-17

**Authors:** Jianhan Zhang, Xu Han, Liang Ma, Shuhui Xu, Yihan Lin

**Affiliations:** Center for Quantitative Biology and Peking-Tsinghua Center for Life Sciences, Academy for Advanced Interdisciplinary Studies, Peking University, Beijing 100871, China; The MOE Key Laboratory of Cell Proliferation and Differentiation, School of Life Sciences, Peking University, Beijing 100871, China; Center for Quantitative Biology and Peking-Tsinghua Center for Life Sciences, Academy for Advanced Interdisciplinary Studies, Peking University, Beijing 100871, China; The MOE Key Laboratory of Cell Proliferation and Differentiation, School of Life Sciences, Peking University, Beijing 100871, China; Center for Quantitative Biology and Peking-Tsinghua Center for Life Sciences, Academy for Advanced Interdisciplinary Studies, Peking University, Beijing 100871, China; The MOE Key Laboratory of Cell Proliferation and Differentiation, School of Life Sciences, Peking University, Beijing 100871, China; Center for Quantitative Biology and Peking-Tsinghua Center for Life Sciences, Academy for Advanced Interdisciplinary Studies, Peking University, Beijing 100871, China; The MOE Key Laboratory of Cell Proliferation and Differentiation, School of Life Sciences, Peking University, Beijing 100871, China; Center for Quantitative Biology and Peking-Tsinghua Center for Life Sciences, Academy for Advanced Interdisciplinary Studies, Peking University, Beijing 100871, China; The MOE Key Laboratory of Cell Proliferation and Differentiation, School of Life Sciences, Peking University, Beijing 100871, China

## Abstract

Cell-to-cell variability within a clonal population, also known as non-genetic heterogeneity, has created significant challenges for intervening with diseases such as cancer. While non-genetic heterogeneity can arise from the variability in the expression of specific genes, it remains largely unclear whether and how clonal cells could be heterogeneous in the expression of the entire transcriptome. Here, we showed that gene transcriptional activity is globally modulated in individual cancer cells, leading to non-genetic heterogeneity in the global transcription rate. Such heterogeneity contributes to cell-to-cell variability in transcriptome size and displays both dynamic and static characteristics, with the global transcription rate temporally modulated in a cell-cycle-coupled manner and the time-averaged rate being distinct between cells and heritable across generations. Additional evidence indicated the role of ATP metabolism in this heterogeneity, and suggested its implication in intrinsic cancer drug tolerance. Collectively, our work shed light on the mode, mechanism, and implication of a global but often hidden source of non-genetic heterogeneity.

## Graphical Abstract

**Graphical Abstract:**
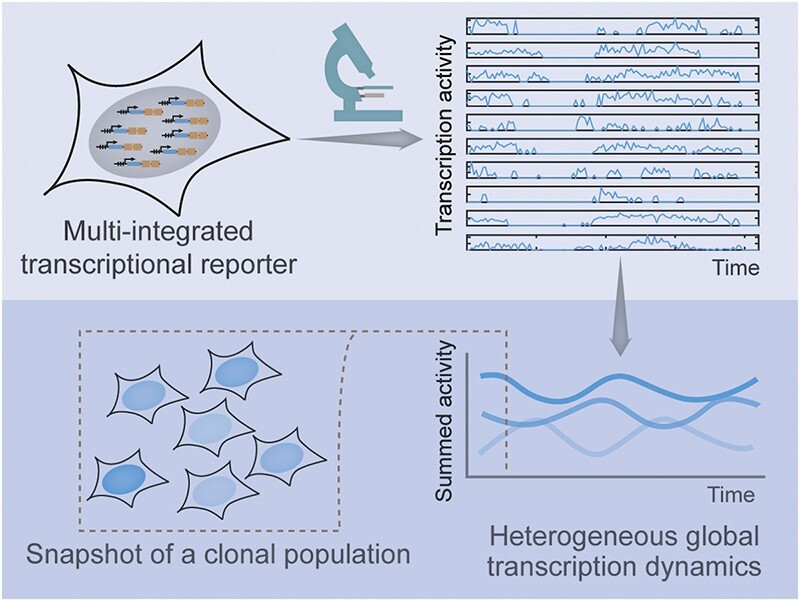

## INTRODUCTION

It has been well established that, although cells in a clonal population share the same genetic background, they are often highly heterogeneous, with genes exhibiting significantly variable expression levels (1–11). Heterogeneous gene expression can lead to phenotypic variations at the single-cell level and has been studied in diverse systems and organisms, from microbial stress response to cancer progression, where it plays critical biological functions and has important clinical relevance (12–17). Thus, understanding the mechanism and function of non-genetic heterogeneity is critical for precisely intervening with diseased cells.

Studies on the source of non-genetic heterogeneity have been largely focused on individual genes (14,18–21), whereby the expression variabilities of specific genes can confer phenotypic heterogeneities that have significant biological implications. For example, heterogeneous expression of a stress protectant allows bet-hedging in microbial cells (22), variability in target genes of pluripotency regulators may underlie developmental priming in early mouse embryos (23), and variable expression level of the secretory leukocyte peptidase inhibitor gene affects cancer clonal dominance (24). Notably, in these examples, the pre-existing, rather than induced, non-genetic heterogeneity can play unexpected roles in affecting the responses to perturbations, highlighting the biological significance of intrinsic clonal variability.

In addition to non-genetic heterogeneity arising from the expression variability of individual genes, recent advances in genomics have implicated the presence of cell-to-cell variability in the expression of the entire transcriptome, as specific perturbations such as transcription factor overexpression or drug treatment can alter the size of the transcriptome by globally modulating gene transcription (25–29). Such global modulation is distinct from gene-specific modulation as it would globally upregulate or downregulate the expression of all genes nonspecifically in a cell. Global modulation of gene transcription has also been inferred by single-cell assays characterizing transcript levels or transcriptional dynamics of multiple genes in the same cell (30–33). However, because of the technical challenges for detecting and analyzing globally modulated transcription in time, its contribution to non-genetic heterogeneity often remains hidden (32). Mechanistically, while cell size (34,35) and mitochondrial level (36,37) have been implicated in the control of global transcription rate, how such factors temporally modulate global transcription in single cells remains largely unclear. Functionally, while it has been implicated that the modulation of transcriptome size can be induced to confer enhanced proliferation or chemotherapeutic resistance for cancer cells (25,26,29), the role of pre-existing cell-to-cell variability in the expression of the entire transcriptome remains elusive.

Temporal analysis of noise or fluctuations leading to non-genetic heterogeneity is necessary for a systematic understanding, as the temporal characteristics of fluctuations can play a pivotal role in determining the mode and function of the resulting clonal heterogeneity (38). As an example, temporal analysis of individual genes’ expression fluctuations has recently revealed that the expression status of a small fraction of genes can be heritable over generations in multiple cancer cell types (39,40), leading to long-lasting (and static) non-genetic heterogeneity in a clonal population and potentially conferring pivotal cancer phenotypes such as drug resistance and metastasis (39,40). Consequently, it would be highly desirable to carry out temporal analysis of fluctuations influencing the expression of the entire transcriptome.

In this work, we set out to temporally analyze the global modulation of gene transcription and decipher the resulting non-genetic heterogeneity in the expression of the entire transcriptome. To probe global transcriptional modulation, we devised an approach based on multi-integrated real-time transcriptional reporters, allowing us to temporally analyze modulated global transcription rate. We found that the instantaneous global transcription rate (i.e. the rate at some instant in time) exhibits temporal fluctuations at the cell cycle timescale, whereas the time-averaged rate is variable among cells, indicating the co-existence of dynamic and static modes of heterogeneity. These observations were corroborated with analyses using public single-cell transcriptome data. Mechanistically, a cell-cycle-dependent ATP metabolic switch likely contributes to dynamic heterogeneity, while heritable differences in the time-averaged metabolic state among cells could give rise to static heterogeneity. Functionally, the heritable global transcription rate was implicated in differential intrinsic drug tolerance in cancer cells. Altogether, our temporal analysis of the global transcription rate yielded key insights into a global source of non-genetic heterogeneity, opening up avenues for broadly analyzing cell-to-cell variability at the global scale.

## MATERIALS AND METHODS

### Cell culture

Human embryonic kidney 293T (HEK293T) and U2OS were used in this study. All cells were cultured with Dulbecco's Minimum Essential Medium (GIBCO, C11995500CP), supplemented with 10% fetal bovine serum (FBS, GIBCO) and 1% penicillin-streptomycin (Sigma-Aldrich). For imaging experiments, normal DMEM was replaced by phenol red-free FluoroBrite DMEM (GIBCO, A1896701) and 10% fetal bovine serum and 1% penicillin–streptomycin were added. All cells were cultured at 37°C with 5% CO_2_ in a humidified incubator.

### Plasmid construction and virus packaging

Molecular cloning was performed according to standard protocols, and plasmids were replicated in DH5α *Escherichia coli*. For the plasmid used for the multi-integrated reporter system, TRE3G promoter sequence, CFP sequence, and 24× PP7 sequence were obtained from existing plasmids in the lab and then inserted into the piggyBac backbone plasmid. For plasmids used for two-color reporter system, endogenous promoters of *ACTB* (−1284 bp to +1052 bp) and *UBC* (−334 bp to +877 bp) were PCR amplified from U2OS genomic DNA and then assembled into 24× MS2 plasmid by Gibson assembly. For cell cycle reporter NLS-Citrine-PCNA, the *PCNA* cassette was constructed by assembly PCR from synthetic DNA oligos, which was then Gibson assembled into lentivirus transfer backbone together with CMV promoter and Citrine cassettes. For ATP biosensor, the ATP sensor sequence was obtained from iATPSnFR1.0 (Addgene #102550), and SV40 NLS sequence was added to the C-terminus to ensure the nuclear localization of the ATP sensor. ATP sensor cassette was synthesized (RUIBO) and inserted into the lentivirus transfer backbone by enzyme ligation. For plasmid validation, 24× PP7 and 24× MS2 repeat sequences were confirmed by enzyme digestion, and all the plasmid sequences were confirmed by Sanger sequencing (RUIBO).

Virus packaging was performed using the three-plasmid system. HEK293T cells at 60–80% plating density were transfected with lentivirus plasmids using polyethyleneimine (PEI). Medium was changed 8 h post-transfection and the culture supernatant containing viral particles was collected two days post-transfection.

### Simulation-assisted reporter design

We simulated the transcriptional dynamics based on a two-state model using the Gillespie algorithm. Genes turn on/off randomly based on the thermodynamic distribution, and transcription only occurs during the on state:


\begin{equation*}OFF\mathop \leftrightarrow \limits^{{\mathrm{\ }}{k}_{on}/{k}_{off}{\mathrm{\ }}} ON\mathop \to \limits^{{\mathrm{\ }}{k}_m{\mathrm{\ }}} nascent\_mRNA\mathop \to \limits^{{\mathrm{\ }}{k}_s{\mathrm{\ }}} mature\_{mRNA}.\end{equation*}

The parameters used in our simulations are: ${k}_{on} = 1.5\ {h}^{ - 1}$, ${k}_{off} = 0.75\ {h}^{ - 1}$,${k}_m = 0.15\ min^{ - 1}$,${k}_s = 0.2\ min^{ - 1}$, where ${k}_{on}$,${k}_{off}$,${k}_m$ were estimated from our imaging experiment of 3-B5-6 cell line in the condition of 0.012 ng/μL doxycycline,${\mathrm{\ }}{k}_s$ was tuned so that the total transcription level is comparable to the experimental results. To model the effect of global fluctuations, we multiplied the parameter ${k}_m$ with a temporal signal representing the global fluctuations, for example, a sine wave function for periodic fluctuations or smoothed random time series for random fluctuations. The rationale for choosing to modulate ${\mathrm{\ }}{k}_m$ is that RNA polymerase elongation can be globally regulated in the cell (37). The amplitude of global fluctuations ranges from 10% to 80%, and the period ranges from 4 to 48 h. The rationale for choosing such an amplitude range or period range was based on experimental measurements (Figure 3H, Supplementary Figure S4D). For a single gene, its transcriptional activity dynamics are quantified as the number of nascent mRNAs being produced over time. Total simulation time lasts 48 h and the sampling interval is 10 min.

The minimum number of reporters (i.e. the number of reporters is designated as n) needed to quantify global fluctuations is measured as the minimal n when the CV^2^ of the sum of n traces (i.e. each reporter gives rise to one nascent transcript count trace) is less than twice the CV^2^ of global fluctuations, so that the magnitude of the global fluctuations is comparable to the intrinsic noise. The rationale for such a criterion is multi-faceted. First, based on experimental results, intrinsic fluctuations are stronger than global fluctuations (Supplementary Figure S4B), i.e. $C{V}_{intrinsic} > C{V}_{global}$. Thus, it is necessary to reduce intrinsic noise by averaging over multiple traces until the strength of intrinsic noise is comparable to that of global fluctuations. Second, it should be noted that the choice of the criterion, i.e. ${CV}_{total}^{2} < {2CV}_{global}^{2}$ or $C{V}_{intrinsic} < C{V}_{global}$, is empirical. This is because only when the intrinsic noise strength is reduced (by averaging) to a level that is comparable to global fluctuations, one can unambiguously detect the temporal waveform of global fluctuations. Please refer to Note S1 for more details.

In Supplementary Figure S3, we simulated different reporter design schemes, and reporters from different regulons were assigned with different promoter switching rate constants, i.e. for promoter X, both ${k}_{on}$ and ${k}_{off}$ are one-fifth of the values above and for promoter Y, both parameters are five-fold of the values above.

### Stable cell line construction

To construct the multi-integrated reporter cell line, plasmids containing TRE3G-CFP-24xPP7 and rtTA were co-transfected into U2OS cells with the piggyBac transposon plasmid by Lipofectamine LTX (Thermo). Single cells containing stably integrated reporter plasmids were FACS-sorted based on the CFP signal into individual culture wells. Many monoclones were infected by lentivirus containing PCP-mCherry and were then screened for the number of nascent reporter transcriptional sites by microscopy. A monoclone with an appropriate number of reporter transcriptional sites was identified and named 3-B5 (third plate, B5 well). To minimize the influence of the heterogenous expression of PCP-mCherry, a monoclone derived from 3-B5 cells was chosen and named 3-B5-6, which was the reporter cell line used in this work. To enable the quantification of cell cycle phase, the 3-B5-6 cell line was infected by lentivirus containing NLS-citrine-PCNA, and the resulting cells were FACS sorted. To enable the quantification of ATP level, the 3-B5-6 cell line was infected by lentivirus containing the aforementioned ATP biosensor, and the resulting cells were FACS sorted. For the construction of dual PP7/MS2 reporter cell line, U2OS cells co-expressing PCP-mCherry and MCP-CFP were FACS sorted after lentivirus infection, and the resulting cell line was transfected with four different plasmids containing TRE3G-YFP-24xPP7, rtTA, endogenous promoter-iRFP-24× MS2 and piggyBac transposase by Lipofectamine LTX. Cells expressing four different fluorescent proteins (mCherry, CFP, iRFP and YFP) were FACS sorted.

### Identification of reporter integration sites

To identify the integration sites of the reporter cell line 3-B5-6, we performed whole genomic DNA sequencing. Genomic DNA of 3-B5-6 was extracted by using a universal genomic DNA kit (CWBio, CW2298). Whole genomic sequencing was performed by GENWIZ. Indels of human genome were mapped by pindel, which yields plasmid integration sites.

### Time-lapse fluorescence microscopy

U2OS cells were seeded into a 24-well glass-bottom plate (Cellvis) 48 h prior to imaging. The culture medium was replaced with phenol red-free medium 8–12 h prior to imaging. Time-lapse images were acquired with an automated inverted microscope Nikon Ti-E with a Plan-Apo 40×/0.85 objective (Nikon). The microscope was equipped with a white-light LED (Lumencor SOLA) and an sCMOS camera (Hamamatsu ORCA-Flash4.0V2). Standard fluorescence microscopy filter cubes (Chroma and Semrock) were used for the acquisition of CFP, GFP, YFP, mCherry and iRFP fluorescence. The sample stage was maintained under 37°C and 5% CO_2_ humidified air in a custom environmental chamber. Multi-channel fluorescence images were acquired every 10 min using a custom Micro-Manager (41) (v1.4) script for 24 to 48 h.

For imaging experiments with the cell line 3-B5-6/Citrine-PCNA, doxycycline was added 12 h prior to imaging to ensure steady-state transcription. The medium was replaced with fresh phenol red-free medium with doxycycline of the same concentration before imaging. For cell cycle blocking experiments, 2 mM thymidine (Sigma-Aldrich, T9250) was added after imaging. Images of mCherry, YPF, CFP and brightfield were captured every 10 min for 48 h. For imaging nascent transcription sites, four z-stacks images with 1.2 μm spacing were acquired to capture loci in different focal planes.

### Image analysis

Fluorescence images were analyzed using a custom MATLAB GUI program that allows automatic analysis as well as manual correction. Images were first background corrected, and segmented to obtain cell masks, which were then tracked across frames to obtain cell trajectories. More specifically, for background correction, the image was fitted using a 2D second-order polynomial function, which was used to remove the background and flatten the original image. The resulting image containing fluorescently-labelled cell nuclei was edge detected using the Sobel operator, dilated, and inner filled to obtain nuclei masks, which were then tracked based on the distances between adjacent frames. The tracks of individual nuclei were manually corrected with the GUI.

For nascent transcription site detection, the z-stacks images were firstly background eliminated, maximum intensity projected, and filtered by a Laplacian of Gaussian filter. The pixel positions of local maxima that pass a pre-defined threshold were regarded as nascent transcription sites. Traces of single loci along time were tracked based on the distance between adjacent frames and were corrected manually. The intensity of individual loci was calculated as sum(signal-bg)/bg, where the signal was defined as the intensity of a 3 × 3-pixel square around the loci pixel and the background (bg) was defined as the median intensity of a 9 × 9-pixel square with a 5 × 5-pixel hollow around the loci pixel. When the loci were below the detection threshold, their intensities would be set to zero. Global transcription rates at specific time points were calculated as the summed intensities of all detected transcription sites in a cell. Mean transcriptional intensities or the number of activated transcription sites were also extracted. Autocorrelation analysis was performed on each cell's global transcription rate trajectory, and the time lag of the first maximum was used as an estimate of the period of the dynamics.

### Cell cycle phase measurement

The cell cycle phase was determined using both the Citrine-PCNA signal and morphological characteristics of the nucleus. More specifically, M phase was identified by the disappearance of nuclear boundaries. During S phase, nuclear Citrine-PCNA aggregates, which can be characterized as the emergence of bright fluorescence puncta or spatially uneven nuclear fluorescence intensities. During the S to G2 transition, Citrine-PCNA gathers to specific genome positions and then dissipates quickly. Because of these characteristics, we defined a parameter PCNA_var_ based on the variance of the PCNA signal as follows:


\begin{equation*}{\mathrm{PCN}}{{\mathrm{A}}}_{{\mathrm{var}}} = \frac{{\max \left( {{I}_{YFP}} \right) - mean\left( {{I}_{YFP}} \right)}}{{mean\left( {{I}_{YFP}} \right)}}.\end{equation*}

Thus, G1/S transition is defined as the first rise of PCNA_var_, and S/G2 transition is defined as the position of max value of PCNA_var_ after G1/S transition.

### Calculation of intrinsic and extrinsic noise

Gene-specific and gene-nonspecific fluctuations (analogous to intrinsic and extrinsic noise) inside a cell were calculated based on the method previously introduced (2,42). Note that we aimed to compare the strengths of fluctuations within a single cell using temporal dynamics of multiple gene loci, which is distinct from typical operations using static snapshots of a population of cells. Because there are more than two dynamic traces in a cell in our experiments, we enumerated all pair combinations in a single cell. For example, if a cell has 10 traces, then 10 × 9/2 = 45 pairs would be enumerated. The intrinsic (gene-specific) noise (${\eta }_{int}$), extrinsic (gene-nonspecific) noise (${\eta }_{ext}$), and total noise (${\eta }_{tot}$) of each pair is defined as follows:


\begin{eqnarray*}\eta _{int}^2 &=& \frac{{\left\langle {{{\left( {{E}_1 - {E}_2} \right)}}^2} \right\rangle }}{{2\left\langle {{E}_1} \right\rangle \left\langle {{E}_2} \right\rangle }},{\mathrm{\ }}\eta _{ext}^2 = \frac{{\left\langle {{E}_1{E}_2} \right\rangle - \left\langle {{E}_1} \right\rangle \left\langle {{E}_2} \right\rangle }}{{\left\langle {{E}_1} \right\rangle \left\langle {{E}_2} \right\rangle }},\nonumber\\ && \eta _{tot}^2 = \frac{{\left\langle {E_1^2 + E_2^2} \right\rangle - 2\left\langle {{E}_1} \right\rangle \left\langle {{E}_2} \right\rangle }}{{2\left\langle {{E}_1} \right\rangle \left\langle {{E}_2} \right\rangle }}.\end{eqnarray*}

Here, ${E}_1$ and ${E}_2$ are the two transcription traces in the chosen pair. The three noise terms were then calculated for all pairs inside each cell and the root mean squares of all pairs were computed as a cell's fluctuation strengths.

### Quantification of dynamic and static heterogeneity using temporal data

Temporal dynamics from our reporter system offer unique opportunities for systematically decomposing variability in the observed signal (i.e. global transcription rate) into static and dynamic components (related to Figure 3H). More specifically, if we define the global transcription rate at a specific time point (t) in a specific cell (c) as ${E}_{t,c}$ (i.e. instantaneous global transcription rate), then the total heterogeneity of the population can be defined as:


\begin{equation*}C{V}_{total} = \frac{{{\mathrm{Std}}{{\left( {{E}_{t,c}} \right)}}_{by\ t,c}}}{{{\mathrm{Mean}}{{\left( {{E}_{t,c}} \right)}}_{by\ t,c}}}.\end{equation*}

To quantify dynamic heterogeneity, we first average rate level across cells and then compute the variability along the cell cycle:


\begin{equation*}C{V}_{dynamic} = \frac{{{\mathrm{Std}}{{\left( {\mathrm{Mean}}{{\left( {{E}_{t,c}} \right)}}_{by\ c} \right)}}_{by\ t}}}{{{\mathrm{Mean}}{{\left( {\mathrm{Mean}}{{\left( {{E}_{t,c}} \right)}}_{by\ c} \right)}}_{by\ t}}}.\end{equation*}

Operationally, to compute the variability along the cell cycle, we need to interpolate the global transcription rate trajectory according to the cell cycle phase measured by the PCNA reporter (i.e. between 0 to 100 percent) in order to average across cells.

To quantify static heterogeneity, we first compute the time-averaged rate level in individual cells, i.e.${\mathrm{\ }}{E}_c = {\mathrm{Mean}}( {{E}_{t,c}} ){\mathrm{\ by\ t}}$. Static heterogeneity can then be calculated as:


\begin{equation*}C{V}_{static} = \frac{{{\mathrm{Std}}{{\left( {{E}_c} \right)}}_{by\ c}}}{{{\mathrm{Mean}}{{\left( {{E}_c} \right)}}_{by\ c}}}.\end{equation*}

The residual heterogeneity is thus:


\begin{equation*}C{V}_{residual} = \sqrt {CV_{total}^2 - CV_{dynamic}^2 - CV_{static}^2} .\end{equation*}

It should be noted that there are other ways to decompose the heterogeneity, for example, $C{V}_{dynamic}$ and $C{V}_{residual}$ could be merged into a single term when $CV_{dynamic}^2$ is defined as the weighted average of individual cells’ $CV_{dynamic}^2$. Yet, we adapted the above decomposition method because we focused on extracting and comparing $C{V}_{static}$ and $C{V}_{dynamic}$, without being influenced by the intrinsic noise in the summed transcriptional activity signal. In other words, in the current decomposition method, the intrinsic noise of the ‘pseudo reporter’ (i.e. the sum of all reporter alleles) is accounted for by the last term, $C{V}_{residual}$. To determine if static heterogeneity simply arises from random fluctuations in gene transcription, we assume that the number of active transcription sites observed during a defined time window within a cell obeys a binomial distribution ${E}_{c\ random} \sim B( {N,p} )$. Here, *N* = 10 total gene loci in our system, and *p* can be calculated by the mean number of active sites over total gene loci *N*. The binomial distribution is then multiplied by the mean transcriptional activity per gene loci to estimate the static heterogeneity arising from random fluctuations.

### PEG-mediated fusion experiment

An entire well of 6-well U2OS cells was first concentrated into 25 μl by centrifugation. The centrifuge tube was kept in a 37°C bath, and 25 μl 50% polyethylene glycol (PEG, MW 8000) was added drop by drop. The tube was then gently shaken to bring the PEG into contact with the cell and remained still for 90 s. 500 μl pre-warmed 37°C Opti-MEM (Gibco) was then added, and the sample tube was further kept in the 37°C bath for 30 min. Cells were then centrifuged and washed with FBS-free culture medium twice and then seeded onto glass-bottom wells for imaging. After seeding, the medium was replaced with a phenol red-free medium containing 0.01 ng/μl doxycycline. Cells were imaged for 24 h with a frame rate of 10 min per frame.

### ATP level quantification

To test whether ATP level correlates with nascent transcriptome or whether ATP level is heritable across generations, nuclear ATP levels in individual cells were measured using the aforementioned ATP biosensor. Nuclear ATP level was quantified as the mean value of GFP intensity inside the nucleus mask.

### Perturbation of ATP metabolism

To perturb ATP metabolism, we used 2-Deoxy-D-glucose (2DG, Sigma-Aldrich, D8375) or oligomycin A (Selleck, S1478) to inhibit different stages of ATP metabolism. For experiments measuring transient responses to inhibitors, cells were prepared as in the time-lapse imaging section, and 5 μM oligomycin or 10 mM 2DG was added after 5–10 h of imaging, and imaging continued for 24 h. For experiments measuring steady-state responses to inhibitors, inhibitors were added 24 h prior to imaging, and imaging continued for 48–72 h. For experiments to measure the transient responses at the S cell cycle phase to inhibitors, 2 mM thymidine was added 24 h prior to imaging.

### Nascent RNA labeling

Nascent RNA labeling was performed by EU (5-Ethynyl Uridine, Abcam ab146642) incorporation. Cells were incubated in the culture medium supplemented with 1 mM EU for 1 h, and were washed by DPBS before fixation in 4% paraformaldehyde. Cellular fluorescence intensity of the ATP sensor and positions of cells were recorded by microscopy before cool ethanol permeabilization. Then incorporated EU was labeled by Click-iT chemistry using Azide Flour 454 dye (Sigma, 760757) or Alexa 488 dye according to manufacturer's instructions (Invitrogen, C10329). The fluorescence of labeled EU from the recoded sample positions was then measured on the microscope.

### Imaging-based intrinsic drug tolerance experiments

To quantify the relationship between global transcription rate and intrinsic drug tolerance, we imaged the 3-B5-6/Citrine-PCNA cell line under 0.01 ng/μl doxycycline (added 12 h prior to imaging) for 24 h before adding indicated chemotherapeutic drug. After drug addition, imaging continued for 72–96 h at the rate of one frame per hour. The death of cells was identified manually by the disappearance of nuclear fluorescence signals as well as the detachment of cells from the surface. In the analysis, the average global transcription rate over the first 24 h without drug was used to quantify the intrinsic global transcription rate in individual cells.

### FACS-based intrinsic drug tolerance experiments

To derive an assay for analyzing intrinsic drug tolerance in a relatively high-throughput format, we need to have enough numbers of cells with either low or high global transcription rates and then subject them to drug treatments, followed by cell survival analysis. We thus resorted to flow sorting of CFP that is expressed under the control of doxycycline induction in the 3-B5-6/Citrine-PCNA cell line. We quantified CFP induction (0.01 ng/μL doxycycline) over a 1-day time course and correlated the intensity of CFP with integrated global transcription rates. By doing so, we found that the two quantities exhibited a maximal correlation at about 12 h post-induction. We concluded that CFP intensity at this time point could be used as a proxy for global transcription rate, and thus sorted based on CFP to obtain cells with either low or high global transcription rates (corresponding to the lowest or highest 20% of CFP intensity). Sorted cells were then plated on 96-well glass bottom plates, allowed for ∼12 h for cell attachment, and drugs were then added at indicated concentrations. Following drug addition, images were taken for at least 12 fields of view to analyze cell numbers, and survival cell fraction at 24, 60 or 72-h post drug addition (depending on the lethality of the drug) was used to compare drug tolerance of cells with low or high CFP.

We took 29 drugs from the Tocriscreen Epigenetics Library (Tocris, Cat. No. 6801) that have been previously used in the literature as tumor-killing drugs (Supplementary Table S1). Out of these drugs, 11 drugs did not display cell killing within three days, one drug caused immediate lethal effect (preventing useful quantification of tolerance), and the remaining 17 drugs displayed cell killing effect within three days (Supplementary Table S1).

### Bioinformatic analysis

For the analysis of single-cell RNA-seq data of U2OS cells, data from GSE146773 and https://github.com/CellProfiling/SingleCellProteogenomics were downloaded, which contained intronic and exonic read counts as well as spiked-in ERCC read counts. We retained cells with ERCC read counts between 100 and 600, and removed genes detected in less than 100 cells. To quantify how the global transcription rate is modulated across the cell cycle, U2OS cells were divided into 14 bins based on the pre-sequenced FUCCI reporter signals, and summed spiked-in normalized total intron read counts were calculated for cells in each cell cycle bin for three different sets of genes, i.e. low expression genes (bottom 30%), medium expression genes (30%-70%), and high expression genes (top 30%). To infer potential regulators of global transcription rate, we used summed spiked-in normalized total intron read counts in individual cells as a ‘target gene’, and used all genes’ normalized exon read counts as ‘regulators’ for inputs of GEINE3 (v1.14.0). Gene ontology analysis of the top 100 genes from GEINE3 was performed by clusterProfiler (v4.0.2). For the analysis of single-cell RNA-seq data of A2780 cells, raw sequencing data from Wang, 2021 (GSE162256) was downloaded and processed. To map the A2780 data, we used Trim Galore (v0.5.0) to remove adaptors and used STAR (v2.5.3a) to map to STAR index generated using spiked-in sequence containing hg38 GENCODE v34 genome. TPM calculator (v0.0.3) was used to compute intronic and exonic read counts, which were then normalized by individual cells’ spiked-in read counts. Because three spiked-in sequences in this dataset varied by 100 times in read counts, we used the spiked-in sequence with the highest expression for normalization. A2780 cells with spiked-in reads larger than half a million or with lower than 10^5^ read counts were filtered out, so were genes detected in less than 5 cells.

For the analysis of drug tolerance using cancer cell line datasets, both gene expression profiles (RNA-seq, 21Q3 release) and drug sensitivity data (Drug sensitivity AUC (Sanger GDSC2) and Drug sensitivity IC50 (Sanger GDSC2)) were downloaded from the Cancer Dependency Map website (https://depmap.org/portal/download/). A dataset containing 597 cancer cell lines and 153 drugs was obtained. Note that not all cell lines have drug sensitivity data for all drugs. Using the expression matrix for the 597 cell lines, we performed hierarchical clustering analysis of the top 100 genes from GEINE3 based on expression correlation, and used genes in the largest co-expression module (*n* = 38 genes) to calculate the modulation index in each cell line, defined as the median expression level of this module. For individual drugs, correlation analysis between drug tolerance measure (AUC or IC50) and modulation index across cell lines was performed.

## RESULTS

### Analysis of public scRNA-seq data implicated the presence of non-genetic heterogeneity in the global transcription rate

While it has been implicated by recent single-cell transcriptome studies that the total amount of mRNAs in a single mammalian cell can be variable within a clonal cell population (43,44), it is unclear whether such non-genetic heterogeneity in transcriptome size represents heterogeneity in the rate of global transcription or heterogeneity in the rate of global mRNA degradation, as the total mRNA abundance is determined by the rate of global transcription together with the rate of global mRNA degradation. Addressing this question would be critical for understanding the cause and consequence of non-genetic heterogeneity in transcriptome size (i.e. total mRNA abundance).

**Figure 1.**
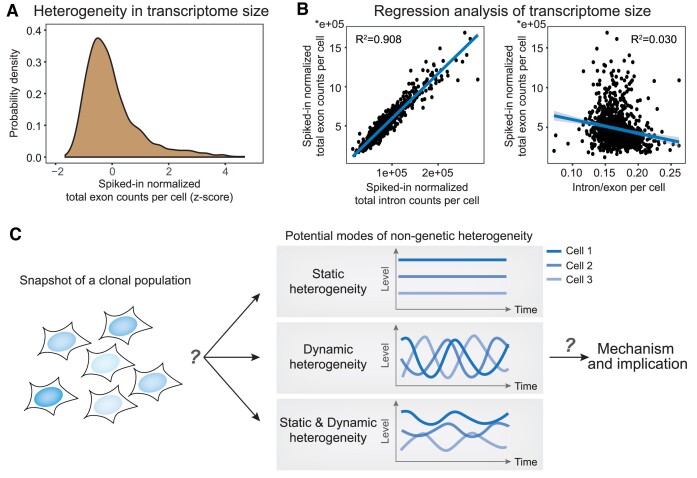
Non-genetic heterogeneity in the global transcription rate. (A, B) Quantification of cell-to-cell variability in transcriptome size using spiked-in normalized single-cell RNA-seq data. (**A**) Distribution of spiked-in normalized total exon counts in single U2OS cells (*n* = 986 cells). Single-cell RNA-seq data from Mahdessian (45) was used (see Materials and Methods for details). (**B**) Scatter plots showing total exon count per cell versus total intron count per cell (left) or the ratio of total intron count over total exon count (right). Total intron count was used as a proxy of the rate of global transcription, while intron/exon ratio is a proxy for global mRNA degradation rate. See Supplementary Figure S1A for explanations. (**C**) Analyses of snapshot single-cell RNA-seq data provoked questions regarding the mode, mechanism, and implication of cell-to-cell variability in the global transcription rate in a clonal population (i.e. non-genetic heterogeneity).

To explore this, we resorted to public single-cell transcriptome data of the human osteosarcoma U2OS cell line with spiked-in RNAs (45), using which we could better estimate the transcriptome size per cell compared to data without spiked-ins (43,44,46). We found that, indeed the estimated transcriptome size displays a large cell-to-cell variability (Figure 1A; Materials and Methods). To investigate the potential cause of such variability, we first analyzed the rate of global transcription in individual cells by leveraging the intronic reads in RNA-seq data, which represent unspliced pre-mature RNAs (47,48) (Supplementary Figure S1A). Importantly, intronic reads can be used as a proxy of the nascent RNA synthesis rate and have been widely used in the analysis of gene transcription kinetics (47–51). We thus used intronic read counts (spiked-in normalized) summing over all genes in each cell as a proxy of the rate of global transcription, and found that the total intronic reads yield a high explanatory power for the transcriptome size (i.e. total exon read count) (Figure 1B, left). We next estimated the rate of global mRNA degradation in individual cells using the ratio of total intronic read count over total exonic read count in a cell (Supplementary Figure S1A), analogous to previous gene-level estimations (47,51). It should be noted that such estimations are built on steady-state assumptions (Supplementary Figure S1A). We found that this rate yields a much weaker explanatory power compared to the global transcription rate (Figure 1B, right). Similar observations were made for another cell line (Supplementary Figure S1B–D). These results indicate that compared to heterogeneity in the global degradation rate, the heterogeneity in the global transcription rate can better explain the heterogeneity in transcriptome size.

Thus, it appears that the global transcription rate is highly heterogeneous across cells, which is an often-hidden source of non-genetic heterogeneity for mammalian cells, as most studies focused on cell-to-cell variability at the level of specific genes. Many aspects regarding the heterogeneity in global transcription rate remain elusive; for example, whether such heterogeneity is dynamic (i.e. heterogeneity arising from dynamic fluctuations) or static (i.e. heterogeneity arising from co-existence of cells with different states), how it is modulated, and what relevance it might have (Figure 1C, Supplementary Figure S1E).

### Simulations guided the design of a reporter system that enables dynamic analysis of global transcription rate

To address these questions, it would be necessary to temporally analyze the global transcription rate in individual cells, because snapshot-based methods such as single-cell sequencing cannot distinguish between dynamic and static modes of heterogeneity. We thus aimed to develop an imaging-based reporter system to analyze global transcription rate dynamics in single cells.

**Figure 2.**
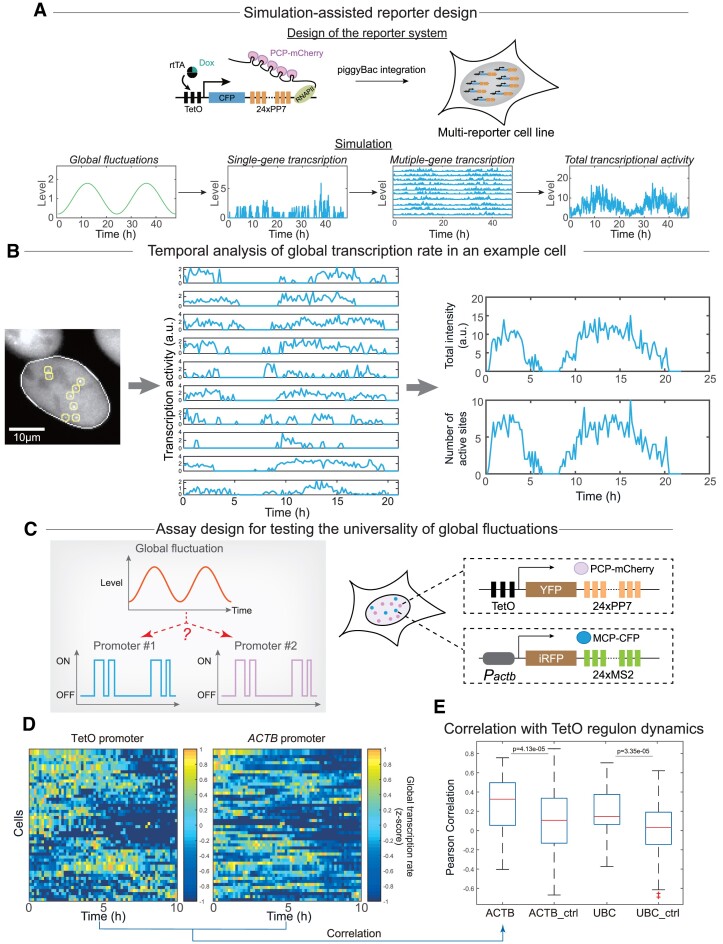
Developing a reporter system for the temporal analysis of global transcription rate. (**A**) Design schematic (top) and in silico validation of the multi-integrated reporter system (bottom). See Materials and Methods and Supplementary Figure S2 for details on simulations. (**B**) Time-lapse imaging of the reporter cell line allows temporal analysis of global transcription rate. An example cell labeled with identified nascent transcriptional sites (left), temporal transcriptional activity dynamics of these sites (middle), and two different quantifications of global transcription rate dynamics (right) were shown. Example snapshots of the example cells were shown in Supplementary Figure S3C. See also Supplementary Video S1. (C–E) Assay for testing whether the observed fluctuations are regulon-specific or shared between regulons. In this assay, two orthogonal RNA-binding protein-based transcriptional reporter systems were used to report the transcriptional dynamics of two different regulons in the same single cells (**C**). Transcriptional activity dynamics of two separate regulons (i.e. PP7-based reporters driven by TetO promoter and MS2-based reporters driven by the promoter of ACTB gene) were quantified in individual cells by summing over the transcriptional activities of detected loci of each regulon (**D**). Correlation between transcriptional dynamics of different regulons in the same single cells was significantly higher than the correlation computed using scrambled cell identities (**E**). Two separate cell lines (i.e. pTetO-PP7/pACTB-MS2 and pTetO-PP7/pUBC-MS2) were used for the quantification (*n* = 51 and 33 cells, respectively). *P*-value was from Welch's *t*-test.

Analyzing global transcription rate dynamics requires capturing instantaneous global transcription rates. However, it is almost impossible to quantify time-lapse global transcription rate by measuring all genes in a single cell. We thus proposed to measure a proxy of global transcription rate by using a reporter system based on multi-integrated transcriptional reporters (Figure 2A top) (52–54). These reporters can report real-time transcriptional activity because the RNA stem-loops on the nascent transcripts would recruit GFP or mCherry-labeled RNA binding proteins such that nascent transcription sites would appear as bright fluorescent foci due to the local enrichment of RNA binding proteins.

To illustrate the rationale behind such an approach, we needed to first discuss the stochasticity involved in gene transcription. The transcriptional dynamics of a gene are typically described by a two-state model whereby the promoter switches stochastically between ON and OFF states, and RNAs are synthesized during the ON state, leading to stochastic bursts of RNA production (Supplementary Figure S2A). The stochasticity in transcriptional dynamics can arise from gene-specific fluctuations (such as the noise inherent to the transcriptional reactions), as well as from gene-nonspecific fluctuations (such as fluctuations in the cellular state that affect many genes) (2,42). Because gene-specific fluctuations are temporally uncorrelated between genes while gene-nonspecific fluctuations are shared across genes, summing transcriptional activity dynamics from multiple genes could in theory, remove gene-specific fluctuations while maintaining gene-nonspecific fluctuations. And as illustrated in the cartoon, the multi-gene-summed activity can serve as a proxy of the global transcription rate, allowing capturing the global modulation of transcriptional activities (Supplementary Figure S2A).

We next used computer simulations to validate the multi-reporter approach and to estimate how many copies of the reporter are sufficient for quantifying the global transcription rate. To do so, we simulated the transcriptional dynamics of genes using two-state models in the presence of both gene-specific and gene-nonspecific (global) fluctuations. Gene-nonspecific fluctuations were implemented by assuming a fluctuating cellular biochemical environment that globally influences the rate of nascent RNA production for all genes (Materials and Methods). To illustrate the effect of both sources of fluctuations, we simulated the transcriptional dynamics of a gene under various global fluctuations, and found that the modulation by global fluctuations can be challenging to identify by looking at a single trace (Supplementary Figure S2B). By summing multiple reporter genes, we could successfully recover the globally modulated transcriptional activity (Figure 2A bottom). We further found that ∼10 copies of the reporter gene would be necessary for capturing global transcription rate dynamics when the global fluctuations are in a sinusoidal waveform, and the fluctuation amplitude is ∼70% of the reporter expression level (Supplementary Figure S2C; Materials and Methods). We demonstrated that this result is generally robust to variations in the underlying kinetic model (e.g. when including two elongation reaction steps to account for the complexity in the RNA polymerase elongation dynamics (55–57) (Supplementary Figure S2D), or when increasing or decreasing the promoter switch rates (Supplementary Figure S2E)) and the waveform of global fluctuations (Supplementary Figure S2F).

### Multi-integrated transcriptional reporters allowed for temporally analyzing global transcription rate dynamics

Based on the in-silico results, we next aimed to construct and test the multi-reporter system experimentally. Our system is based on a synthetic transcriptional reporter containing CFP and RNA binding stem-loops (24× PP7) (54), driven by doxycycline-inducible promoter TRE3G. When transcribed, the 24 copies of the PP7 stem-loop would recruit stably expressed PCP-mCherry protein, allowing the visualization of nascent transcription sites as fluorescent puncta (Figure 2A, top). This reporter was then multi-integrated into U2OS cells using PiggyBac transposase. The resulting monoclones were screened by imaging, and a monoclone with ∼12 visible nascent transcription sites was chosen, which was then sequenced to map reporter integration sites. We identified nine integrated reporter copies across seven different chromosomes by genome sequencing (Supplementary Figure S3A), yet there are likely more reporter copies that were not captured by sequencing (Supplementary Figure S3B).

Using the reporter cell line, we performed time-lapse imaging of single cells and quantified the fluorescence intensity traces of individual nascent transcription sites (i.e. individual fluorescent puncta in the nucleus). Intriguingly, when summing transcriptional activity traces from all reporter loci in a single cell, we were able to observe large fluctuations in the summed activity dynamics (Figure 2B, Supplementary Figure S3C, Supplementary Video S1). This observation is consistent with the in-silico result that summing over a sufficient number of reporters can recover shared global fluctuations. However, such global fluctuations could result from fluctuations either inside the cell (e.g. changes in the intracellular environment) or outside the cell (e.g. changes in the culture condition). To distinguish between the two sources, we compared the summed transcriptional activity traces from cells in the same culture environment and found that global fluctuations were unsynchronized across cells, indicating that the fluctuations are cell-intrinsic (Supplementary Figure S3D). Together, these results indicated that the reporter system could capture global transcription rate dynamics in single cells. It should be noted that the summed transcriptional activity dynamics only provide an estimate of the global transcription rate dynamics, i.e. such a reporter system focuses on capturing the temporal pattern and the relative magnitude, instead of the absolute magnitude, of the global transcription rate.

Nevertheless, the above data prompted several questions regarding the characteristics of global transcription rate dynamics. First, because the reporter genes are driven by the same promoter (i.e. doxycycline-inducible promoter, TRE3G), it is critical to discern whether the observed dynamics are indeed global, instead of regulon-specific. Second, because the transcriptional dynamics of individual reporter genes are subjected to both gene-specific and gene-nonspecific fluctuations, it is necessary to compare the relative strength of the two noise sources inside cells. Additionally, because the magnitude of fluctuations should depend on the level of transcription, it is necessary to quantify such dependency. Third, besides characterizing the magnitude of fluctuations in global transcription rate dynamics, it is also critical to profile their temporal characteristics.

First, to discern if the fluctuations in the summed activity dynamics are global or regulon-specific, we needed to test whether genes driven by different promoters (i.e. different regulons) would be influenced by the same fluctuations. To do so, we constructed a cell line with two different types of transcriptional reporters (by using PP7 and MS2 systems (52–54)), each driven by different promoters (e.g. TRE3G and *ATCB* promoter, respectively) (Figure 2C, Materials and Methods). If the fluctuations are regulon-specific, the dynamics of the two types of reporters would not exhibit a significant correlation, and vice versa if the fluctuations are global. As a negative control, reporters from different cells should not correlate. By performing two-color time-lapse imaging, we successfully captured the transcriptional dynamics of two different types of reporters using two separate RNA-binding protein systems (Supplementary Figure S3E, Figure 2D). We found that reporters from two regulons in the same cell exhibited a significantly positive correlation. In contrast, reporters from different cells were not correlated (Figure 2E). This result is consistent with the presence of global fluctuations influencing regulons nonspecifically. Characterization of another cell line containing reporters driven by TRE3G and *UBC* promoters provided additional support for this conclusion (Figure 2E).

To further address whether it is sufficient to capture global fluctuations using reporter genes of a single regulon or if reporter genes from different regulons are necessary to remove potential ‘regulon-specific’ effect, we computationally compared different reporter designs, with reporter genes from one to three regulons (Supplementary Figure S3F). We found that the capability to capture the global fluctuations depended on the number of reporter genes, rather than the number of regulon types used (Supplementary Figure S3G). Importantly, for the design with two types of regulons (which is analogous to the two-regulon experiments above), the correlation between the summed transcriptional activities of the two regulons was markedly lower than the correlation between each regulon's summed transcriptional activities and the global fluctuation waveform (Supplementary Figure S3G), consistent with the relatively low correlation between regulons observed in the experiments (Figure 2E).

We next addressed how such global fluctuations are compared to gene-specific (i.e. intrinsic) fluctuations in terms of the magnitude of fluctuations. To do so, we decomposed fluctuations in transcriptional activity into gene-specific and gene-nonspecific components by adapting the concept from the two-color reporter assay (2,42). More specifically, based on activity traces from any two reporter loci in a cell, we used mean-normalized variance and covariance between two signals to estimate gene-specific and gene-nonspecific sources of fluctuations, respectively (Supplementary Figure S4A, Materials and Methods). By performing such calculations across all pairs of reporter loci in a cell and using the averaged results as cell-specific fluctuation strength, we found that the strengths of both fluctuations decrease as transcriptional activity increases (i.e. doxycycline concentration increases) and that gene-specific fluctuations are more substantial than gene-nonspecific fluctuations across different doxycycline conditions (Supplementary Figure S4B). Thus, genes transcribing at lower activity levels are more susceptible to global fluctuations compared to those transcribing at higher activity levels. Moreover, because gene-specific fluctuations are much stronger than gene-nonspecific fluctuations, these results implicate that gene-specific control of transcriptional bursting (i.e. the source of gene-specific fluctuations) mainly contributes to the strength of stochasticity in individual genes’ transcriptional activities, whereas gene-nonspecific fluctuations mainly contribute to the temporal organization of transcriptional activities (Supplementary Figure S4C), highlighting the importance of temporal analysis for studying gene-nonspecific fluctuations.

We further investigated the temporal characteristics of global transcription rate dynamics by performing auto-correlation analysis, which detects potential periodicity. We computed auto-correlation functions of individual cells’ summed transcriptional activity traces under various doxycycline concentrations, and estimated the periodicity by measuring the distance between the first two peaks in the auto-correlation function. The results showed that global fluctuations consistently exhibit a ∼12 h periodicity across different transcriptional activity levels (Supplementary Figure S4D, E), indicating that global fluctuations are potentially driven by or coupled to periodic cellular signals.

Together, using the multi-integrated reporter system, we revealed that the gene-nonspecific fluctuations affecting reporter transcription are global instead of regulon-specific, and that such fluctuations are much weaker than gene-specific fluctuations inside a cell. Importantly, the reporter systems allowed us to capture global transcription rate dynamics by averaging out gene-specific fluctuations, revealing the potentially periodic nature of such dynamics.

### Global transcription rate contains both static and dynamic components of non-genetic heterogeneity

While global transcription rate fluctuates temporally within a single cell, the mode of the resulting non-genetic heterogeneity within a clonal cell population remains to be further examined. This would allow us to address whether cells can access distinct or overlapping global transcription rates, and whether these rates are heritable between generations, which are necessary for further dissecting the mechanism and implications.

**Figure 3.**
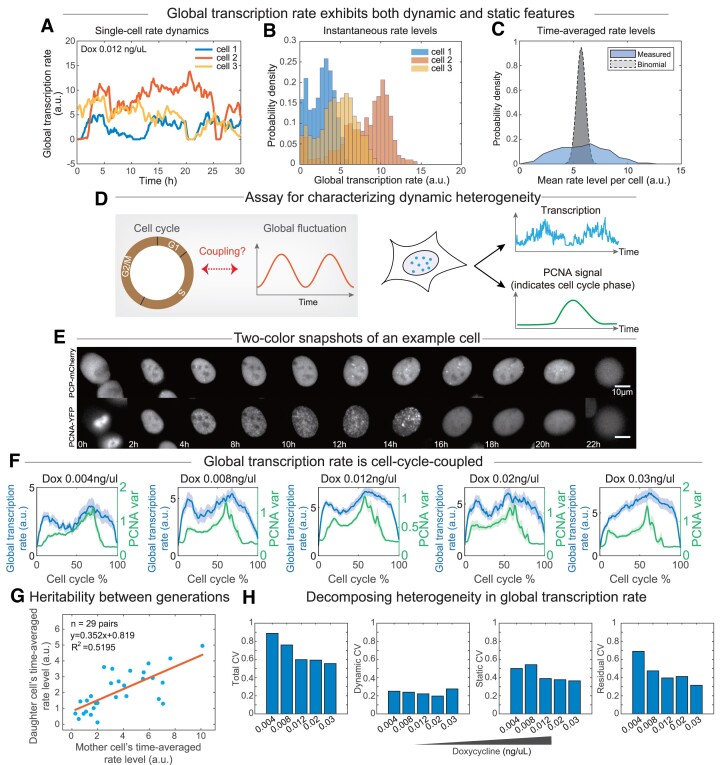
Multi-integrated real-time transcriptional reporters allowed characterizing non-genetic heterogeneity in the global transcription rate. (A–C) Inferring the mode of heterogeneity using temporal data from the reporter system. Global transcriptional rate dynamics from three example single cells acquired under indicated condition (**A**) were used to construct distributions of instantaneous rate levels (**B**). The distribution of time-averaged rate levels in individual cells (n = 51 cells) was compared to a control distribution (i.e. binomial, see Materials and Methods) (**C**). (D–F) Characterizing the dynamic mode of heterogeneity in individual cells. To address whether global transcription rate is coupled to the cell cycle (left, **D**), simultaneous quantification of the reporter system and the cell cycle is needed (right, D). Snapshots from simultaneous imaging of transcription and cell cycle marker PCNA-YFP at indicated time points post mitosis in an example cell (**E**). Population-averaged global transcription rate dynamics and the cell cycle signal (quantified by variance in nuclear PCNA signal) were plotted for five indicated conditions (**F**). (**G**) Scatter plot showing time-averaged global transcription rate levels in pairs of mother and daughter cells. (**H**) Clonal variability in global transcription rate level from all cells in snapshots (characterized by the coefficient of variation, or total CV; 1st panel) can be decomposed into dynamic variability along the cell cycle time (characterized by dynamic CV; 2nd panel), static or inter-cellular variability (characterized by static CV, 3rd panel), and the residual CV (last panel). See Materials and Methods for details.

We first aimed to discern between static and dynamic modes of heterogeneity using dynamic data. More specifically, we asked whether the global transcription rates in individual cells are persistently and statically different (i.e. static heterogeneity) or whether different cells are dynamically accessing a shared distribution of rates (i.e. dynamic heterogeneity). By examining the global transcription rate dynamics of different cells (Figure 3A), we found that while global transcription rate fluctuates within each cell, the accessible rates of different cells come from overlapping distributions (Figure 3B). Moreover, the mean of each cell's rate (i.e. time-averaged global transcription rate) is much more widely distributed when compared to the scenario where all gene loci were independently and randomly regulated (Figure 3C, Supplementary Figure S5A, Materials and Methods). These results indicate the co-existence of both dynamic and static heterogeneity, whereby global transcription rates in individual cells fluctuate around distinct mean rates but with overlapping instantaneous rates.

The above findings prompted us to further characterize the dynamic aspect of the heterogeneity by depicting how the global transcription rate temporally fluctuates in individual cells. Because the global fluctuations exhibit a ∼12-h periodicity (Supplementary Figure S4D), which is close to half of the cell cycle length, we speculated that global transcription rate could be dynamically coupled to the cell cycle (Figure 3D). To explore this, we constructed a cell cycle sensor based on PCNA fusion protein (Citrine-PCNA) (58), allowing us to identify G1, S and G2/M phases based on the Citrine-PCNA puncta formed during DNA replication (Figure 3E). By imaging reporter transcription and citrine-PCNA signals simultaneously in the same single cells, we found that global transcription rate is indeed cell-cycle-coupled, eliciting two pulses in a cell cycle (Figure 3F, Supplementary Figure S5B). More specifically, the rate level peaked at the G1 phase and late S or early G2/M phase, respectively, and reached the trough at the mid-S phase. Note that when reporters transcribed strongly (i.e. under the highest doxycycline concentration), the two pulses are not as obvious (Figure 3F), consistent with the earlier finding that genes with higher transcriptional activities are less susceptible to global fluctuations (Supplementary Figure S4B).

To further dissect the coupling between the cell cycle and global transcription rate, we asked whether perturbing the cell cycle would affect global transcription rate dynamics (Supplementary Figure S5C). We thus used thymidine to block DNA replication and found that the rate dynamics no longer exhibited a pulsatile behavior, but instead sustained at a relatively high level when cells were blocked at the S phase (Supplementary Figure S5D, E). This result suggested that the troughing of global transcription rate is coupled to S phase progression, and that the global transcription rate is actively modulated during the cell cycle.

Given the co-existence of both dynamic and static heterogeneity, we next focused on the static aspect of the heterogeneity. Note that static heterogeneity is used here to refer to variability between cells, while dynamic heterogeneity is used to refer to temporal variability within specific cells. While we have demonstrated that individual cells can have distinct time-averaged global transcription rates (Figure 3C, Supplementary Figure S5A), indicative of static heterogeneity, it remains to be addressed whether such static heterogeneity is heritable across generations. We thus computed the correlation between cell-cycle-averaged global transcription rates of mother and daughter cells, and observed a significant positive correlation (Figure 3G). Note that 3-B5-6/citrine-PCNA cell line (Materials and Methods) was used in this experiment (see Note S2 for more details). In other words, the global transcription rates in both mother and daughter cells would temporally fluctuate around mean levels that are significantly correlated. Thus, the global transcription rate appears to be heritable between successive generations, which would contribute to the persistence of clonal heterogeneity. It should be noted that such heritability is likely transient, i.e. only across neighboring generations, as the *R*^2^ value is not very high.

To further quantify heterogeneity, we next compared the contributions from dynamic and static aspects of heterogeneity to non-genetic heterogeneity in global transcription rate. Non-genetic heterogeneity is typically measured in a static population of cells (e.g. by flow cytometry, image snapshots, or scRNA-seq), which can be regarded as the total heterogeneity in a population and can be quantified by the coefficient of variation (CV). Our temporal measurements allowed deconvolving the total heterogeneity into ones contributed by different aspects, including dynamic and static heterogeneity (Materials and Methods). To quantify the degree of static heterogeneity, we computed the coefficient of variation (CV) of the population-averaged and cell-cycle-normalized global transcription rate trajectory (Figure 3H, Materials and Methods). To quantify the contribution of dynamic heterogeneity, we computed the CV of the time-averaged rates from all individual cells. We found that compared to dynamic heterogeneity, static heterogeneity yields a larger contribution to the total heterogeneity in global transcription rate (Figure 3H), and that the relatively large residual CVs indicated the presence of fluctuations intrinsic to global transcription rate (that were not averaged away by the multi-reporter assay). Reassuringly, simulations of a population of cells with intrinsically different mean global transcription rates supported key findings of the experimental results (Supplementary Figure S5F–I): (i) both static and dynamic heterogeneities can be consistently extracted when using different reporter designs (i.e. either the strength or the copy number of the reporter); (ii) the residual CV primarily represents the intrinsic noise of the estimated global transcription rate as it could be largely reduced by increasing the copy number.

Because cell size has been implicated in global transcription control (34,35), we next asked whether the observed heterogeneity in global transcription rate relates to cell size. To do so, we used the size of the nucleus as a correlate of cell size (59) (Supplementary Figure S6A) and characterized how time-averaged nuclear size correlates with time-averaged global transcription rate, and how nuclear size dynamically changes over the cell cycle in individual cells. Interestingly, we found that time-averaged nuclear size displays a statistically significant (but a relatively low magnitude) correlation with time-averaged global transcription rate across single cells only when the doxycycline concentration is low (Supplementary Figure S6B), indicating that nuclear size differences could contribute to the static heterogeneity of the global transcription rate for lowly transcribing genes. Meanwhile, we found that the dynamic patterns of nuclear size along the cell cycle are distinct from those of global transcription rate (Supplementary Figure S6C). Therefore, further temporal studies are necessary to substantiate the role of cell size in global transcription.

### Single-cell transcriptome analysis supported the presence of a hybrid mode of heterogeneity in the global transcription rate

Thus far, we have established that global transcription rate is heterogeneous in a clonal population of cells, and such heterogeneity is contributed both by static and dynamic components. Because these results were obtained using temporal data from the reporter system, we next sought to employ single-cell transcriptome data of the same cell type to validate key conclusions.

**Figure 4.**
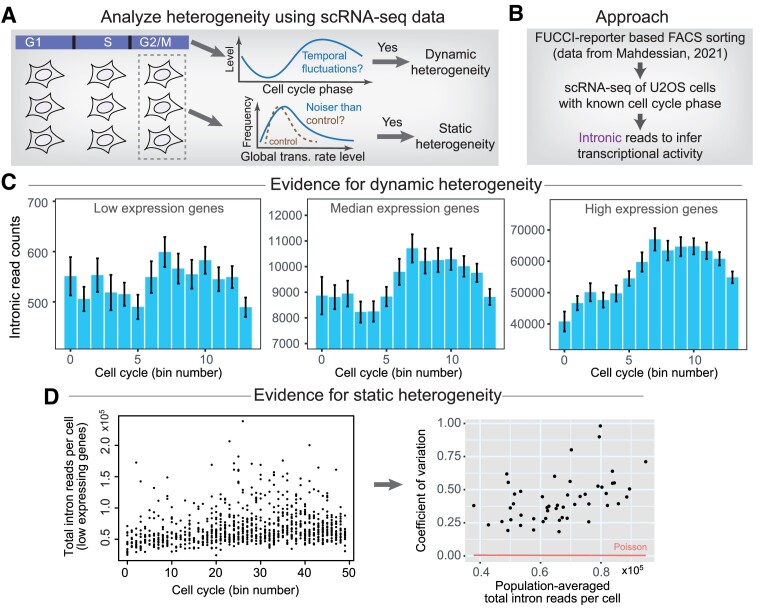
Single-cell transcriptome data supported a hybrid mode of heterogeneity in the global transcription rate. (A–D) Quantifying non-genetic heterogeneity in global transcription rate using spiked-in normalized single-cell transcriptome data (public data). To detect dynamic heterogeneity, cells from the different cell cycle phases could be separately averaged to obtain the dynamics along the cell cycle. To detect static heterogeneity, variability in cells from the same cell cycle phase could be compared to the variability of a control distribution (**A**). To achieve this, cell-cycle-sorted single-cell RNA-seq data from Mahdessian, 2021 was used (**B**) (with spiked-in RNAs). Intronic read counts summing over genes of differential expression levels in individual cells were calculated, and results from cells along different cell cycle bins were averaged (*n* = 33–79 cells in each bin) (**C**). The variability of total intron counts (of low expressing genes) of cells in each cell cycle bin was compared with the variability of a Poisson distribution of cells with the same mean count (**D**). Note that in (D), the CVs from Poisson distributions are between 0.003 and 0.005 (which appeared to be zero in the plot).

We therefore resorted to the earlier dataset that contains transcriptome measurements of cell-cycle-sorted U2OS cells (45). This dataset would allow us to test whether cells from different cell cycle phases would exhibit variable global transcription rates (i.e. dynamic heterogeneity), and if there are intercellular variabilities within cells from the same cell cycle phases (i.e. static heterogeneity) (Figures 4A, B).

By using summed intronic read counts (which are spiked-in normalized) as a proxy of global transcription rate and by binning cells based on cell cycle phases, we calculated the averaged global transcription rates along the cell cycle (Materials and Methods). The resulting ‘pseudo dynamics’ of the global transcription rate exhibited two activity pulses within one cell cycle (Figure 4C), which reassuringly agrees with the temporal dynamics from our reporter system (Figure 3F). Moreover, when comparing genes transcribing at different rates across the transcriptome, we found that low-expression genes were more susceptible to the modulation (Figure 4C). These results supported the presence of dynamic heterogeneity and further substantiated our conclusion that gene transcriptional activity is indeed globally and temporally modulated in a gene-nonspecific manner.

To quantify static heterogeneity, we needed to compute intercellular variability among cells within the same cell cycle phase and compare such variability to variability arising from Poisson fluctuations. We thus placed cells into 50 bins along the cell cycle, and within each bin, the CV of the total intronic read counts was calculated (Figure 4D). To calculate CV from Poisson fluctuations, we modeled the detection of each gene's introns as a Poisson process, and the CV was then estimated as the inverse of the squared root of total intron counts. By doing so, we found that intercellular variability is considerably above Poissonian variability (Figure 4D), supporting the existence of intercellular variability in the global transcription rate.

Together, analyses of single-cell transcriptome data provided additional evidence supporting the co-existence of dynamic and static heterogeneity in global transcription rate, highlighting the perplexing nature of such non-genetic heterogeneity.

### Perturbation assays suggested the role of ATP metabolism in conferring the non-genetic heterogeneity of global transcription rate

**Figure 5.**
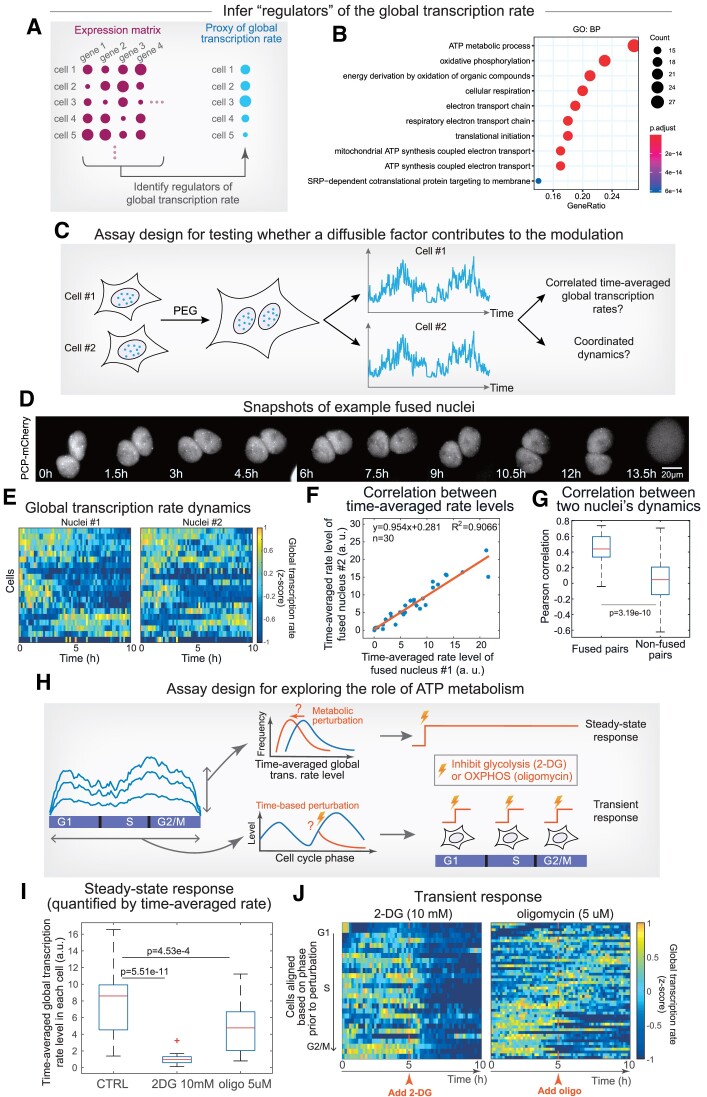
Evidence indicating the role of ATP metabolism in global transcription rate modulation. (A, B) Inferring potential regulators of the global transcription rate using single-cell transcriptome data. Single-cell expression matrix (normalized exon counts of genes by cells) was used as an input of GEINE3 for the inference of regulators for the global transcription rate (**A**). Results from gene ontology analysis of the top 100 potential regulator genes (**B**). (C–G) Cell fusion assay for testing if gene transcription is globally modulated by a diffusible factor. Design of the cell fusion assay (**C**). Snapshots showing two PEG-fused nuclei at indicated time points (**D**). Heatmaps showing z-scaled global transcription rate dynamics of two fused nuclei (**E**). Scatter plots showing two nuclei’ time-averaged rate levels in each single cell (**F**). Box plots showing the correlations between the dynamics of fused nuclei pairs and between the dynamics of non-fused nuclei pairs (i.e. control) (*n* = 30 nuclei pairs and *P*-value from Welch's *t*-test) (**G**). (H–J) Responses of global transcription rate to perturbations in ATP metabolism. Assay includes two different experiments to measure the steady-state responses and transient responses to inhibition of glycolysis (using 10 mM 2-DG) or inhibition of oxidative phosphorylation (5 μM oligomycin) (**H**). Boxplots showing single-cell time-averaged global transcription rate levels under three conditions (*n* = 36, 20, 23 cells from left to right, *P*-values from Welch's *t*-test) (**I**). Heat maps showing z-scaled global transcription rate dynamics in individual cells before and after indicated perturbations (*n* = 27, 49 cells from left to right) (**J**). Cells were vertically aligned according to their cell cycle phases at the time of perturbation. See also Supplementary Figure S8A-D for additional quantifications.

To further decode such heterogeneity, we next focused on its mechanistic underpinnings. We began by leveraging single-cell transcriptome data to search for potential regulators whose expression levels can explain the differences in global transcription rates among single cells. In doing so, we assumed that both inter- and intra-cellular variabilities in global transcription rate are potentially driven by changes in specific regulators that lead to changes in the global cellular state. To detect such regulators, we used a gene regulatory network inference package based on random forest regression (i.e. GEINE3 (60)) to rank genes based on their explanatory powers for global transcription rate (Figure 5A). Intriguingly, the top 100 genes are highly enriched for biological processes related to ATP metabolism (Figure 5B), indicating that ATP metabolism may be playing a key role in modulating global transcription rate.

Following this lead, we asked whether ATP level correlates with global transcription rate in single cells. We used an ATP biosensor (61) to measure nuclear ATP levels and subjected the same cells to a pulse of nucleoside analog 5-EU (5-ethynyl uridine) to measure instantaneous global transcription activities (Materials and Methods). The rationale to use 5-EU labeling for measuring global transcription activity is that the transiently incorporated 5-EU can represent the instantaneous transcription activity of all genes in the cell and that the cell line containing the ATP biosensor is technically challenging to install the PP7-based reporter system (i.e. it would require generating a monoclonal cell line). Importantly, we demonstrated that the amount of incorporated 5-EU correlated with the summed PP7 signals in the reporter cell line (Supplementary Figure S7A, B), providing a key support for using either 5-EU labeling or PP7-based reporter system for quantifying global transcription activity. Cells with ATP biosensor were stained to label the incorporated EU, after which we simultaneously quantified ATP levels and global transcription activity (estimated by the amount of incorporated EU) in the same cells with two-color fluorescence imaging (Supplementary Figure S7C). Intriguingly, we found that the two signal levels are significantly correlated (Supplementary Figure S7D), consistent with a hypothesis that metabolites produced during ATP metabolism (such as ATP) could globally modulate gene transcriptional activity nonspecifically, leading to heterogeneity in the global transcription rate.

To test this hypothesis, several key issues needed to be addressed. First, it is important to validate whether the global transcriptional modulation is mediated via a diffusible mechanism (e.g. through metabolites) or a non-diffusible mechanism (e.g. through chromatin dynamics). Second, if ATP metabolism plays a key role, it is necessary to test whether perturbations to ATP metabolism would impact the dynamics and the level of global transcription rate. Third, because the global transcription rate is heritable between successive generations, it is critical to deciphering whether ATP metabolic state is also heritable.

We first tested whether a diffusible mechanism could be responsible for global transcriptional modulation. We reasoned that if metabolites from ATP metabolic reactions (that occur in cytoplasm or mitochondria) diffuse into the nucleus to modulate transcription globally, two nuclei embedded inside the same cytoplasm should exhibit correlated global transcription rate levels and coordinated global transcription rate dynamics (Figure 5C). By using PEG to fuse the membranes of two cells and imaging the resulting fusion cells to quantify global transcription rate dynamics (Figures 5D, E), we reassuringly found that the time-averaged global transcription rates are significantly correlated between two nuclei in individual fusion cells (Figure 5F), and that their dynamics are also highly coordinated (Figure 5G), supporting that a diffusible mechanism potentially mediates the global modulation of gene transcription in the nucleus.

We next perturbed ATP metabolism and quantified the impacts on the global transcription rate. Because ATP metabolism involves both glycolysis and oxidative phosphorylation, we reasoned that inhibiting either stage of ATP metabolism should lower the steady-state level of global transcription rate (Figure 5H). When applying 2-DG and oligomycin to separately inhibit glycolysis and oxidative phosphorylation and imaging cells under perturbation for at least one cell cycle, we found that time-averaged global transcription rates were significantly reduced in either perturbation (Figure 5I). Notably, global transcription rate was more susceptible to glycolysis inhibition, indicating a more important role of glycolysis than oxidative phosphorylation in the modulation.

Moreover, because the global transcription rate is dynamically modulated during the cell cycle, we dynamically inhibited different stages of ATP metabolism along the cell cycle and quantified the immediate responses, allowing us to establish the dynamic role of ATP metabolism on global transcription during the cell cycle (Figure 5H). By doing so, we unexpectedly found distinct dynamic roles of glycolysis and oxidative phosphorylation on global transcription rate modulation (Figure 5J). More specifically, inhibition of glycolysis led to a significant reduction in global transcription rate, which is independent of the cell cycle (Figure 5J left, Supplementary Figure S8A, B). In contrast, inhibition of oxidative phosphorylation had a cell-cycle-dependent effect, as it reduced global transcription rate only after the S phase (Figure 5J right, Supplementary Figure S8C, D). These intriguing perturbation outcomes are consistent with a picture that the global transcription rate switches to an increased dependency on oxidative phosphorylation for ATP metabolism during the mid-S phase while maintaining a steady dependence on glycolysis throughout all cell cycle phases.

Based on these observations, we hypothesized that blocking cells at S phase would sensitize the response to the inhibition of oxidative phosphorylation, but not to the inhibition of glycolysis. We thus used thymidine to block cells prior to the addition of inhibitors, and found that when blocked at the S phase, global transcription rates were indeed more sensitive to the addition of oligomycin (Supplementary Figure S8E–G) compared to the scenario without blocking (Figure 5J right, Supplementary Figure S8C, D). In contrast, the responses to the inhibitor of glycolysis did not display such a contrast (compare Supplementary Figure S8H-J with Figure 5J, left and Supplementary Figure S8A, B). Such a differential sensitivity to glycolysis inhibition versus oxidative phosphorylation inhibition can be further visualized when plotting all the results together (Supplementary Figure S8K).

Thus, these dynamic perturbation results implicated that a cell-cycle-coupled switch in ATP metabolism (i.e. switch to an increased dependency on oxidative phosphorylation) modulates global transcription rate dynamics, whereby the decreasing energy supply (or other metabolites from ATP metabolism) prior to the switch drives the decrease in global transcription rate, which is then boosted upon switching (giving rise to the second pulse of global transcription rate).

Lastly, a long-time imaging experiment using the ATP biosensor was performed, and the result showed that the time-averaged ATP level was heritable across successive generations (Supplementary Figure S8L), supporting the potential role of ATP metabolism in conferring heterogeneity in global transcription rate.

In sum, these results suggest an intriguing role of ATP metabolism in both the temporal modulation of global transcription rate as well as the modulation of time-averaged global transcription rate inside single cells, highlighting the potential function of metabolic dynamics in conferring non-genetic heterogeneity in mammalian cells. However, it should be noted that drug perturbations to metabolism could cause alerted metabolism of other metabolites, and we cannot exclude the roles of such alterations in global transcription rate.

### Heterogeneous global transcription rate is associated with the intrinsic drug tolerance of cancer cells

Non-genetic heterogeneity of cancer cells has attracted growing interest, as it has been implicated in chemotherapeutic resistance, metastasis, and tumor microevolution (12,16). While global transcriptional amplification has been observed in cancer cells, most studies focused on the role of induced global transcriptional amplification (25,26,29), rather than the pre-existing heterogeneity in the global transcription rate. Nonetheless, as we have established that the global transcription rate is heterogenous at steady state (without perturbation) and is transiently heritable across cell generations, it remains elusive whether such pre-existing intrinsic non-genetic heterogeneity may have functional implications.

**Figure 6.**
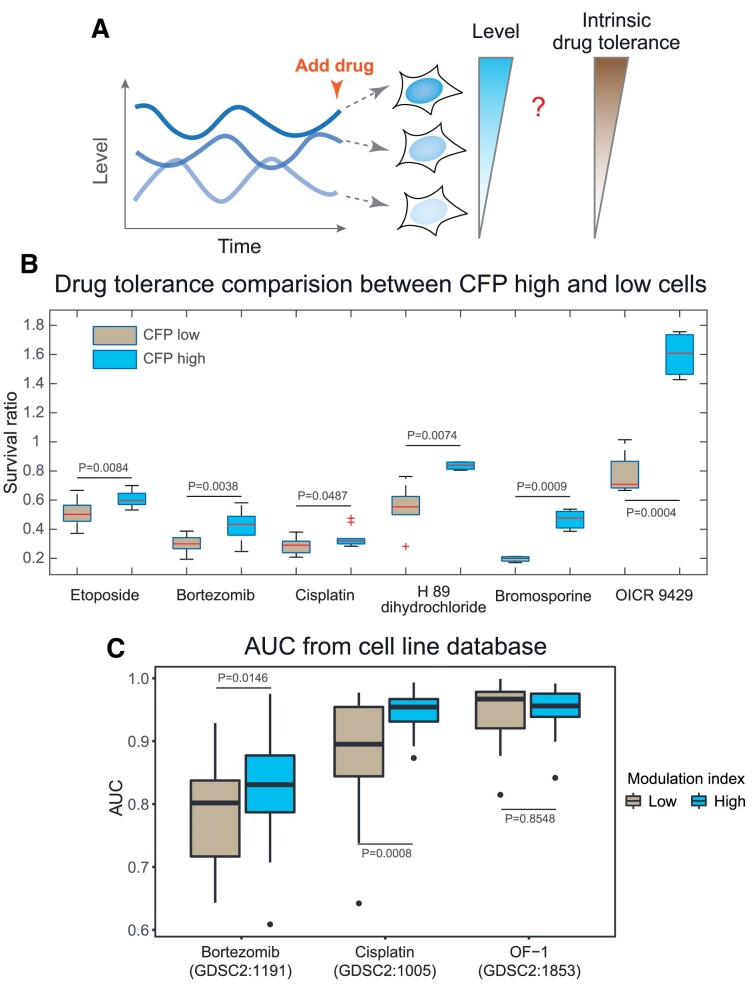
The functional implication of intra-clonal global transcription rate variability in intrinsic drug tolerance. (**A**) Hypothetical cartoon. Heterogeneous and heritable time-averaged global transcription rates in single cells could confer intrinsic differences in drug tolerance. (**B**) Boxplots showing cell fraction survived after indicated drug treatments for U2OS cells with low or high CFP levels. Cells were sorted based on CFP intensity, subjected to drug treatments, and then quantified by imaging (see Materials and Methods for details and Supplementary Table S1 for information on concentrations). *n* = 3–10. *P*-value from Welch's *t*-test. (**C**) Boxplots showing AUC values (characterizing drug tolerance level) from GDSC2 database for cell lines with low or high modulation indexes. *n* = 39 and 30 cell lines with low or high modulation indexes, respectively. *P*-value from Welch's *t*-test.

Because of the earlier study indicating the potential role of global transcriptional amplification in drug resistance (29) as well as the recent interests in the role of pre-existing non-genetic heterogeneity in chemotherapeutic resistance (30,62,63), we set out to characterize the functional implication of heterogeneous global transcription rates in intrinsic drug tolerance. We reasoned that because individual cells have variable time-averaged transcription rates prior to drug treatment, such variability could relate to differential drug tolerances for different cells (Figure 6A). To test this, we carried out a single-cell drug tolerance assay to quantify the relationship between drug tolerance and global transcription rate (Supplementary Figure S9A).

More specifically, we imaged cells with the reporter system for 24 h prior to the addition of a commonly used chemotherapeutic drug, etoposide (targeting topoisomerase II), and continued the imaging for another two days (Supplementary Figure S9B, Materials and Methods). By quantifying the intrinsic global transcription rates in individual cells and measuring the survival time of the corresponding cells in the presence of etoposide, we found that the intrinsic global transcription rate exhibited a statistically significant positive correlation with the survival time (Supplementary Figure S9C), yet the magnitude of the correlation is relatively moderate, possibly because of both the biological noise in the system as well as the technical challenge in accurately determining the time of cell death. Intriguingly, similar results were observed for two additional chemotherapeutic drugs, bortezomib (inhibiting the 26S proteasome) (Supplementary Figure S9D) and cisplatin (DNA damaging) (Supplementary Figure S9E).

Due to the limited throughput of imaging-based survival assay, we next aimed to test more drugs by developing an assay with higher throughput. We enriched cells with low or high global transcription rates by FACS sorting after ∼12 h of doxycycline induction (Supplementary Figure S9F, Materials and Methods) and then compared their susceptibility to drugs. Reassuringly, this new assay recapitulated results from the imaging assay, i.e. cells with higher global transcription rates displayed higher tolerance to the three drugs tested above (the leftmost three drugs in Figure 6B, Supplementary Figure S9G). Note that 3-B5-6/Citrine-PCNA cell line (Materials and Methods) was used in this experiment (see Note S2 for more details). We then used this assay to study 26 more drugs, and among the total of 29 drugs, only 17 drugs display appropriate cell-killing effects (Materials and Methods, Supplementary Table S1). Among these 17 drugs, six were significantly better tolerated by cells with higher global transcription rates (Figure 6B), and the rest (except one) were not differentially tolerated (Supplementary Figure S9H).

These results are generally consistent with the picture that cancer cells with high global transcription rates exhibited increased tolerance to certain anti-tumor drugs, yet the difference among drugs remains to be explored. While this finding reconciles with the recent report that drug-induced (i.e. non-intrinsic) global transcriptional amplification is relevant to drug resistance (29), our result highlights the potential implication of pre-existing (i.e. intrinsic) heterogeneous global transcription rates in drug tolerance. Note that these results were based on the 3-B5-6/Citrine-PCNA cell line, which was constructed by lentiviral transduction of a cell cycle reporter (i.e. Citrine-PCNA) into a monoclonal transcriptional reporter cell line (i.e. 3-B5-6). While we cannot rule out the potential caveats arising from the viral transduction step, we believe it is reasonable to assume the monoclonality of the transcriptional reporters, as Citrine-PCNA introduced to the 3-B5-6 monoclone is irrelevant to the transcriptional reporter system (see Note S2 for more details).

To further explore this implication, we resorted to cancer cell line databases, including CCLE and GDSC (64–67), where the bulk transcriptome profiles and drug sensitivity profiles have been characterized for an extensive collection of cell lines and drugs. The rationale is that different cell lines may possess differential global transcription rates and thus variable intrinsic drug sensitivities (Supplementary Figure S10A, B). We sought to derive a marker gene set whose expression level could be used to estimate global transcription rates in specific cell lines. Using the top 100 genes identified in our previous analysis (that can best explain global transcription rate in the single-cell sequencing dataset) (Figure 5B) and the expression profiles of the cancer cell lines from the database, we identified the largest co-expressing gene module consisting of 28 genes. We used this gene set to compute a modulation index for each cell line as a proxy for the extent of global transcriptional modulation (Supplementary Figure S10C, Materials and Methods).

We first investigated drugs in the database that overlap with the 17 drugs that were experimentally measured. Reassuringly, for the three overlapping drugs, the drug sensitivity data from the database agree with our experiments (Figure 6C). More specifically, for two of the overlapping drugs (bortezomib and cisplatin), cell lines with high modulation indexes displayed higher tolerances compared to cell lines with low modulation indexes, consistent with our experimental observations (Figure 6C, B, Supplementary Figure S9C–E); and for the third overlapping drug (OF-1), no differential drug tolerance was observed for both the database and our experiment (Figure 6C, Supplementary Figure S9H).

We next asked whether drug sensitivity would display an overall correlation with modulation index in the database. Using the drug sensitivity data for camptothecin (a topoisomerase inhibitor) as an example, we found that the modulation indexes in different cell lines exhibit a significant positive correlation with either of the two drug sensitivity measures, IC50 and AUC (Supplementary Figure S10D, E). Importantly, similar results were observed for most of the drugs targeting different signaling mechanisms (Supplementary Figure S10F, G), indicating that the modulation index of a cancer cell line relates to the degree of intrinsic multi-drug tolerance of the cell line. Therefore, this result further indicates the potential biological implication of global transcription rate modulation. It should be noted that the above analyses using the public cancer cell line databases were built on the assumption that the modulation index correlates with the extent of global transcriptional modulation, and thus the implications based on such analyses would need further investigations.

## DISCUSSION

**Figure 7.**
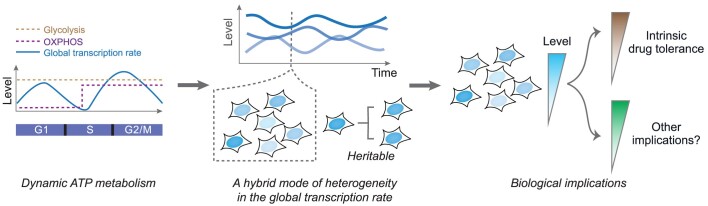
A global source of non-genetic heterogeneity: mode, mechanism, and implication. Cartoons illustrating a working model for a global source of non-genetic heterogeneity in mammalian cells. Our results showed that ATP metabolism likely plays a key role in conferring heterogeneity in the global transcription rate (left). In this model, cell cycle phase-dependent alteration in oxidative phosphorylation activity may result in dynamic global transcription rates along the cell cycle, with the time-averaged rates being modulated by mean levels of ATP metabolism. Intra-clonal variability in global transcription rate thus displays features of both dynamic and static modes of heterogeneity, with the static heterogeneity being heritable across successive generations (middle). Notably, such pre-existing heterogeneity between clonal cells could lead to differential responses to perturbations, and may thus have implications in intrinsic drug tolerance, among others (right).

It has been well established that the expression levels of individual genes are often highly heterogeneous in a clonal population, and such heterogeneity can play pivotal roles in a range of biological processes. In contrast, due to technical challenges, cell-to-cell variability in the expression of the entire transcriptome has remained largely unexplored. We addressed this challenge by integrating time-lapse imaging with transcriptome analysis, and uncovered that gene transcription is globally and temporally modulated in a gene-nonspecific manner, resulting in heterogeneous and heritable global transcription rates in individual cells (Figure 7). Intriguingly, our single-cell drug tolerance assay and analyses of cancer cell line datasets indicated the potential implication of such heterogeneity in conferring intrinsic drug tolerance, underscoring the importance of studying non-genetic heterogeneity at the global scale.

Deciphering the sources of fluctuations is critical for understanding heterogeneity in a clonal population. Because global (gene-nonspecific) fluctuations can convolute with gene-specific fluctuations to confer heterogeneity in a clonal population, it has thus been highly challenging to quantify the global source of fluctuations (18). Our reporter system was rationally designed to leverage the correlated nature of global fluctuations among genes, allowing us to temporally analyze fluctuations in global transcription rate that affect all genes nonspecifically (Figure 2). Compared to temporal analysis using our reporter system, although single-cell transcriptome analysis can only provide a snapshot of cells and thus cannot distinguish between modes of heterogeneity, it can still be used to infer the presence of transcriptome size heterogeneity (43,44). Thus, as a complement to our temporal analysis, it would be helpful to systematically quantify absolute single-cell transcriptomes across diverse conditions and cell types to determine the pervasiveness of heterogeneity in the global transcription rate. However, it is important to note that most existing single-cell transcriptome datasets cannot be used for analyzing such heterogeneity, as protocols without using external spiked-ins do not measure absolute transcriptomes.

While genes are often specifically regulated by upstream regulators to cope with various environments, increasing evidence has suggested that the activities of many genes can also be globally modulated during important cellular processes, such as cancer progression and stem cell differentiation (68,69). Our finding provides quantitative evidence that global modulation is not only highly dynamic, but also highly cell-specific, displaying features of both static and dynamic heterogeneity (Figure 3 and Supplementary Figure S5). Notably, mitochondrial ATP production has been shown to be a global influencer of gene transcription (37), and it was thought that the cell-to-cell variability in global gene transcription is contributed by the variability in the mitochondrial mass (37). However, while mitochondrial mass variability may provide an explanation for the static component of heterogeneity we observed in the global transcription rate, several lines of evidence from our study point to the potential role of dynamic ATP metabolism in shaping such heterogeneity (Figure 5 and Supplementary Figure S8), underscoring the emergent roles of cellular metabolism in the temporal organization of cellular transcriptome. Yet, because most of the key evidence was derived from perturbation experiments using 2-DG or oligomycin, we could not exclude the potential roles of other metabolites perturbed in the experiments. Therefore, how ATP metabolism dynamically modulates global transcription rate and what specific and fundamental molecular mechanisms are involved in the modulation require further investigations, so as the role of additional factors or processes other than ATP metabolism. More generally, the interplay between metabolism, cell size, and global transcription rate modulation at the single-cell level necessitates further studies.

At the functional level, dynamic and cell-specific modulation of the transcriptome may bias cells towards specific states or fates while maintaining dynamic plasticity. As we have demonstrated, cells with high global transcription rates were able to survive longer when challenged with three different chemotherapeutic drugs (Figure 6). While this observed correlation implicates the potential role of heterogeneous global transcription rate and is consistent with the result from the cell line data analysis, it remains elusive what molecular entities are responsible for the longer drug survival and what other functional roles the heterogeneity may play. Nonetheless, our result highlights the necessity to carefully examine the consequences of variability within a clonal population, and adds to the growing list of evidence supporting the important, but often hidden, role of non-genetic heterogeneity in cancer cells (12,24,30,62).

## Supplementary Material

gkad666_Supplemental_Files

## Data Availability

All relevant data have been included in this manuscript. Additional data are available from the corresponding author upon request.
