# Supplementary material for: Deciphering a global source of non-genetic heterogeneity in cancer cells
Source: Nucleic Acids Res. 2023 Aug 17;51(17):9019–38. doi: 10.1093/nar/gkad666 (PMC10516630; doi:10.1093/nar/gkad666)
Supplement: gkad666_Supplemental_Files [file gkad666_supplemental_files.zip › 2023-7-9-Supplementary Materials.pdf]

Supplementary Materials for

Deciphering a global source of non-genetic heterogeneity in cancer cells

Jianhan Zhang[1,2,#], Xu Han[1,2,#], Liang Ma[1,2], Shuhui Xu[1,2], Yihan Lin[1,2,*]

Table of Contents

Supplemental Notes **1**

Note S1 2

Note S2 4

Supplemental Figures **7**

Figure S1 7

Figure S2 8

Figure S3 10

Figure S4 12

Figure S5 13

Figure S6 15

Figure S7 16

Figure S8 17

Figure S9 19

Figure S10 20

Supplemental Table **21**

Table S1 21

Supplemental Video **22**

Video S1 22

Supplemental Notes

Note S1. The criterion used for determining the minimum number of reporters.

In the Materials and Methods section, we described the method for determining the minimum number of reporters (i.e., n reporters) needed to quantify global fluctuations. We used a criterion whereby the minimum number of reporters was determined by the minimal n when the CV^2 of the sum of n traces is less than twice the CV^2 of global fluctuations, so that the magnitude of the global fluctuations is comparable to the intrinsic noise. To explain this criterion, we here provide a detailed explanation using simple simulations.

To begin the explanation, we needed to define the different types of noise involved. In our model, gene expression level can be described by the following equation:

$$E = E_{mean} + \eta_{global} + \eta_{intrinsic}.$$

Here, $<\eta_{global}> = 0$, $<\eta_{intrinsic}> = 0$, and E_{mean} is a fixed value. In the manuscript, when discussing global fluctuations, we referred to the fluctuations of gene expression when considering global fluctuations only, i.e., $E' = E_{mean} + \eta_{global}$. In this case, the degree of fluctuations in E' was regarded as global fluctuations, i.e., $CV_{global} = Std(\eta_{global})/E_{mean}$.

We first consider a single trace subjected to sinusoidal global fluctuations and intrinsic fluctuations defined by a uniform distribution as in the following equation:

$$E = E_{mean} + \eta_{global} + \eta_{intrinsic} = 1 + \sin(x) + U(-1, 1).$$

Under this scenario, $CV^2_{global} = Var(\eta_{global})/E^2_{mean} = 1/2$ and $CV^2_{intrinsic} = Var(\eta_{intrinsic})/E^2_{mean} = 1/3$. Thus, $CV^2_{total} = CV^2_{intrinsic} + CV^2_{global} = 5/6 < 2CV^2_{global} = 1$, which meets our criteria. In other words, this single trace would capture the temporal waveform of global fluctuations. To test this, we plotted the simulated trace together with the global fluctuation waveform (**Figure SN1**). Obviously, such a single trace captures the temporal waveform of global fluctuations, consistent with the expectation of our analysis.

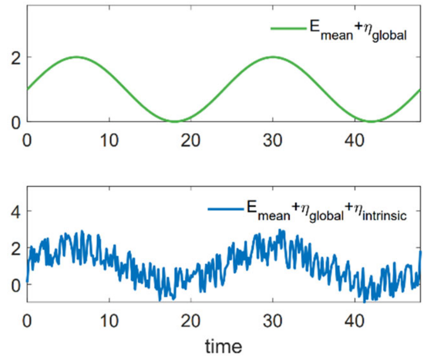

Figure SN1. A single trace could capture the global fluctuation waveform. For this example trace (bottom), it is influenced by both sinusoidal global fluctuations (top) and intrinsic fluctuations defined by a uniform distribution *U(-1, 1)*.

Why a single trace is sufficient? It is important to note that in the particular example above, the magnitude of global fluctuations is larger than that of intrinsic fluctuations, and

thus a single trace already meets our criteria, i.e., "CV^2 of the sum of n traces is less than twice the CV^2 of global fluctuations".

However, in real datasets intrinsic fluctuations are stronger than global fluctuations, i.e., $CV_{intrinsic} > CV_{global}$. This is evident from the analysis in Figure S4B of our manuscript. Therefore, as illustrated in our simulations, ~10 traces are needed in order to capture the temporal waveform of global fluctuations. To better illustrate this notion, we provided simulations of different numbers of traces, from 1 to 10 (**Figure SN2**). By comparing these simulation results, it is evident that when 10 genes were used, it is sufficient to capture the temporal waveform of global fluctuations (**Figure SN2**).

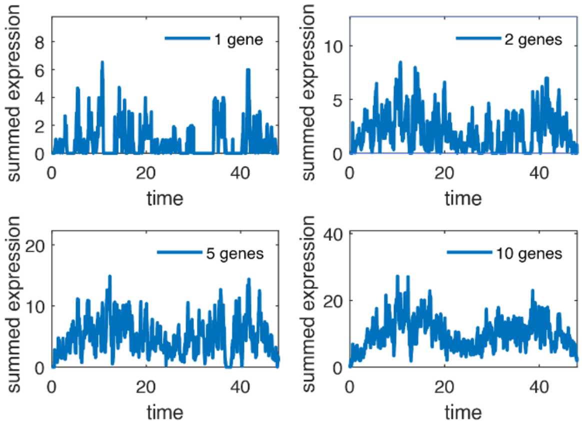

Figure SN2. Summing over different numbers of simulated traces. Same simulations as in Figure S2C were performed and the summed traces were shown for scenarios where different numbers of simulated traces were included. It is evident that intrinsic fluctuations were suppressed when traces from more genes were summed.

We further plotted CVs of intrinsic and extrinsic (global) fluctuations for the data above (**Figure SN3**), i.e., $CV_{intrinsic}$ and CV_{global}. When only one trace was used, CV_{global} was much weaker than $CV_{intrinsic}$, and under such a scenario, one trace was not sufficient for capturing the temporal waveform of global fluctuations (**Figure SN2**). In contrast, when 10 genes were used, $CV_{intrinsic}$ is comparable to but lower than CV_{global} (**Figure SN3**), and the summed trace was able to capture global fluctuations (**Figure SN2**).

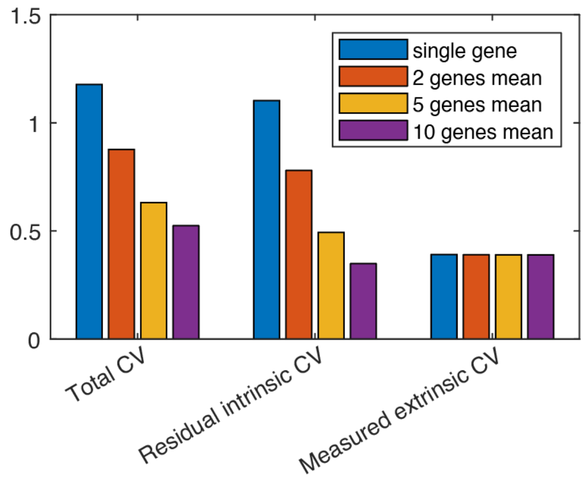

Figure SN3. Quantification of the magnitude of fluctuations when summing over different trace numbers.

By comparing the two scenarios above, the number of traces increases from 1 to 10, and $CV_{intrinsic}$ is correspondingly reduced by ~70%, reaching a level that is slightly lower than CV_{global}. In other words, $CV_{total}^2 = CV_{intrinsic}^2 + CV_{global}^2 > 2CV_{global}^2$ when n = 1, and $CV_{total}^2 = CV_{intrinsic}^2 + CV_{global}^2 < 2CV_{global}^2$ when n = 10. Thus, our criterion was met when n = 10 genes.

It should be noted that the choice of the criterion, i.e., $CV_{total}^2 < 2CV_{global}^2$ or $CV_{intrinsic}^2 < CV_{global}^2$, is empirical. This is because that for a single gene's transcriptional dynamics, the strength of intrinsic noise is typically much stronger than that of global noise, and as long as the intrinsic noise strength can be reduced (by averaging) to a level that is comparable to global noise, one can easily detect the temporal waveform of global fluctuations. And if when the strength of intrinsic noise is comparable to that of global noise, no averaging would be needed to detect global fluctuations. In this regard, our criterion provides general guidance for determining the number of reporters needed for averaging and is not limited to specific conditions.

Note S2. Additional experiments characterizing the non-genetic heterogeneity in global transcription rate.

For Figure 3G and Figure 6B, cell lines were based on the monoclonal transcriptional reporter cell line (3-B5-6), which was constructed by piggyBac-mediated integration of multiple transcriptional reporters, followed by monoclonal purification. Then, lentivirus containing Citrine-PCNA reporter was introduced into this monoclonal reporter cell line to allow the quantification of cell cycle phases, whereby viral-transduced cells were sorted to ensure similar expression levels of Citrine-PCNA reporter (note that it is technically almost impossible to perform a second monoclonal purification at this step due to cellular senescence). We think it is a reasonable and acceptable practice to assume that the monoclonality of the transcriptional reporters is maintained in such viral-transduced cells (3-B5-6/Citrine-PCNA), because that 1) the Citrine-PCNA introduced during this step is not related to the function of the transcriptional reporter system, and 2) viral transduction was achieved at low MOI to ensure single-copy integration, and it is unlikely that such an integration event would result in relevant genetic variations.

However, specific concerns could still arise regarding the data in Figure 3G and Figure 6B. First, the observed correlation between pairs of mother and daughter cells (Figure 3G) could arise from genetic heterogeneity caused by viral integration. Second, the observed difference

in drug tolerance could be due to differences in the copy number or the integration site of the lentivirus (instead of associating with the level of the global transcriptional activity, Figure 6B).

To address these and related concerns, we performed two new experiments to ask whether the correlation between pairs of mother and daughter cells, as well as the association between drug tolerance and global transcriptional activity level, could be observed in a clonal population of cells displaying non-genetic heterogeneity. In other words, in these new experiments, we sought to establish that non-genetic heterogeneity in global transcriptional activity in a clonal population of cancer cells could be inherited by daughter cells and that cells with higher global transcriptional activity levels are associated with enhanced drug tolerance.

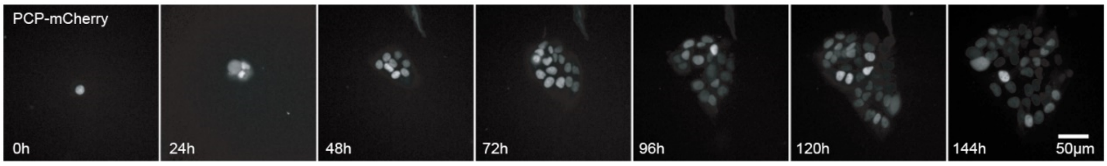

Figure SN4. Snapshots from a 6-day time-lapse fluorescence imaging of a clone of cells growing from a single cell. mCherry fluorescence images of the PCP-mCherry fusion protein at indicated time points were shown. The scale bar indicates 50 μm.

In the first experiment, we performed lineage tracing of clonal cells growing from individual single cells by conducting long-term time-lapse imaging of 3-B5-6/Citrine-PCNA cells (i.e., monoclonal 3-B5-6 cells transduced with lentivirus containing the cell cycle reporter). As shown in the example snapshots (**Figure SN4**), cells were seeded at a relatively low density to enable the long-term tracing of the clonal cell population growing from each single cell. Using such data, we can quantify global transcription rates in each clonal population of cells, and determine the correlation between mother and daughter cells. This can be accomplished by tracing cells in each clonal population and quantifying global transcription rate levels in each pair of mother and daughter cells.

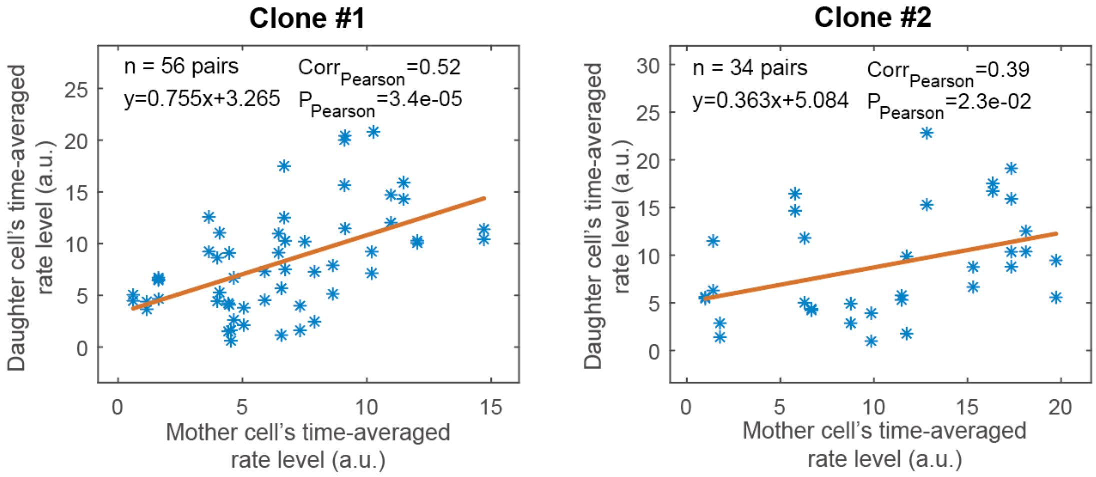

Figure SN5. Two scatter plots showing time-averaged global transcription rate levels in pairs of mother and daughter cells in two different clonal cell populations.

Importantly, we found that in two separate clones, the time-averaged global transcription rate levels were significantly correlated between pairs of mother and daughter cells (**Figure SN5**). Therefore, we conclude that the heritability in the global transcription state can be observed in a clonal population of cells that are genetically identical, greatly substantiating the conclusion from Figure 3G.

In the second experiment, we performed drug tolerance quantifications analogous to the experiment in Figure 6B by using strictly monoclonal cells. More specifically, we used 3-B5 cells, which are monoclonal U2OS cells integrated with the transcriptional reporters (and without the PCP-mCherry protein). This monoclonal cell line was used to construct the subsequent cell lines such as the 3-B5-6 cells. We sorted cells based on the induced CFP intensity and treated low or high CFP cells with etoposide, one of the drugs used in Figure 6B. Reassuringly, we found that cells with high CFP cells displayed significantly enhanced tolerance to the drug, as more cells survived after ~24h of drug treatment (**Figure SN6**). This result is consistent with the picture that the heterogeneous global transcription rates of cells from a clonal population are associated with differential degrees of drug tolerance, providing additional support for the conclusion from Figure 6B.

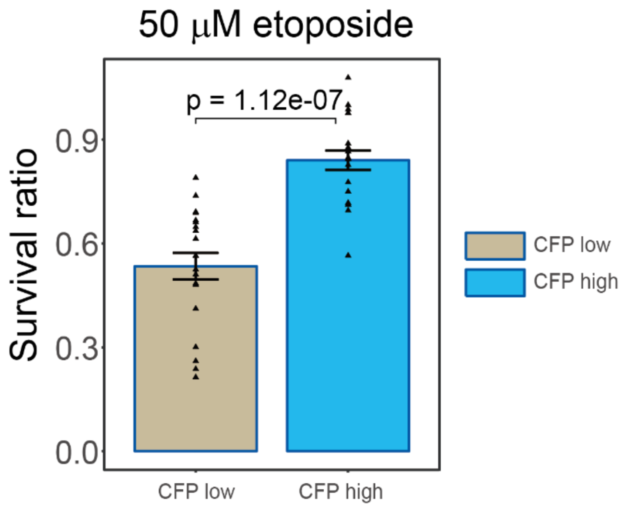

Figure SN6. Bar graph showing cell fraction survived after indicated drug treatment for monoclonal U2OS cells with low or high CFP levels. Cells were sorted based on CFP intensity, subjected to drug treatment, and then quantified by imaging (see Materials and Methods for details). n = 20.

Figure S1

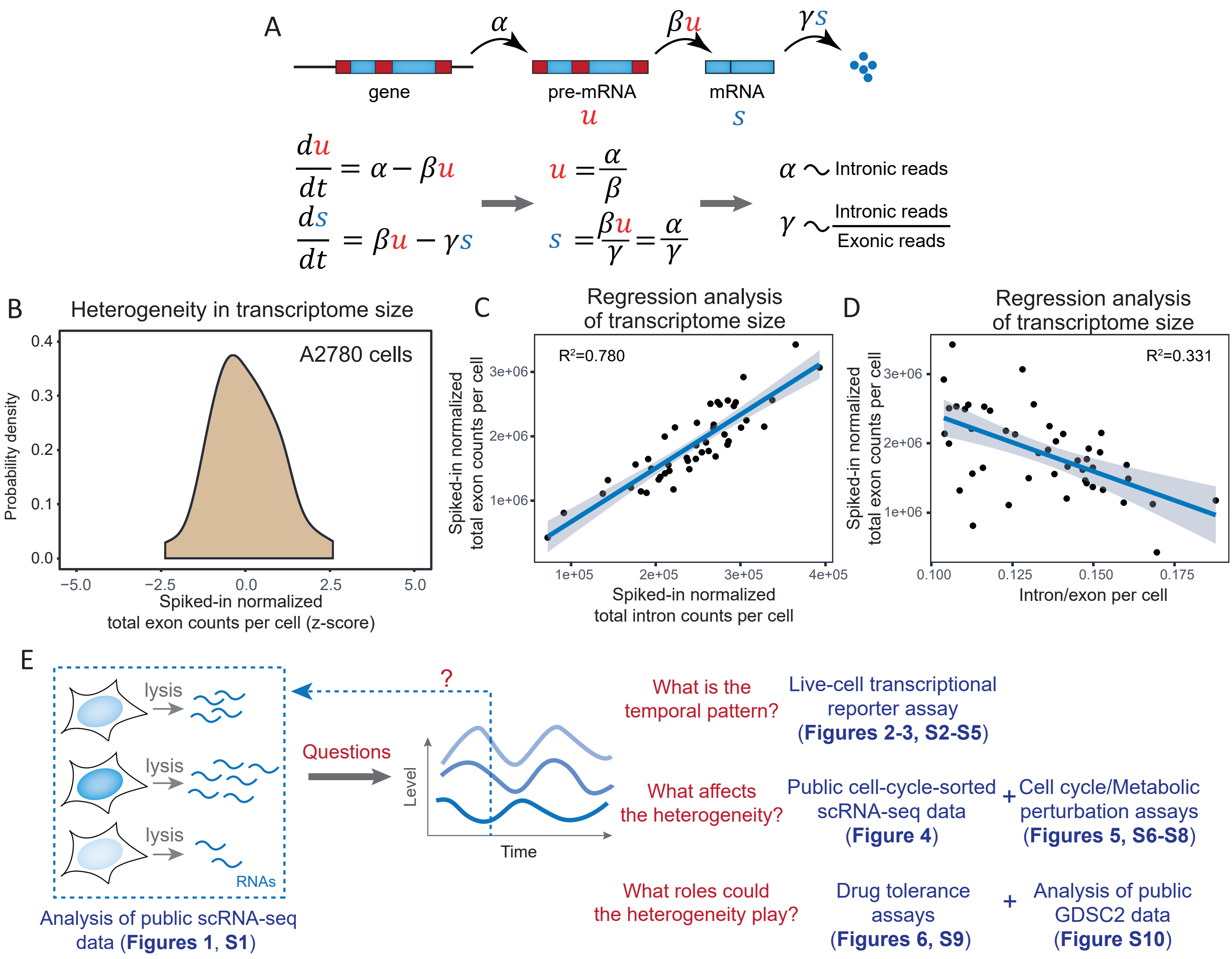

Figure S1 | Characterization of single-cell transcriptome size variability.

(A) Cartoon illustrations of gene expression reactions and the associated ordinarily differential equations. In an RNA-sequencing dataset, intronic reads are typically reads from unspliced pre-mRNAs while exonic reads are typically reads from spliced mRNAs. When assuming a constant splicing rate across cells (which is a potential caveat in this model), the rate of transcription could be estimated using the intronic read counts, while the degradation rate of mRNA could be estimated using the ratio between intronic and exonic reads.

(B) Distribution of spiked-in normalized total exon counts in single A2780 cells (an ovarian cancer cell line, n = 46 cells). Single-cell RNA-seq data from Wang, 2021 was used (see **Materials and Methods** for details).

(C-D) Scatter plots showing total exon count per cell versus total intron count per cell (C) or the ratio of total intron count over total exon count (D). Analogous to **Figure 1B**.

(E) Summary of the logic and assays in the study.

Figure S2

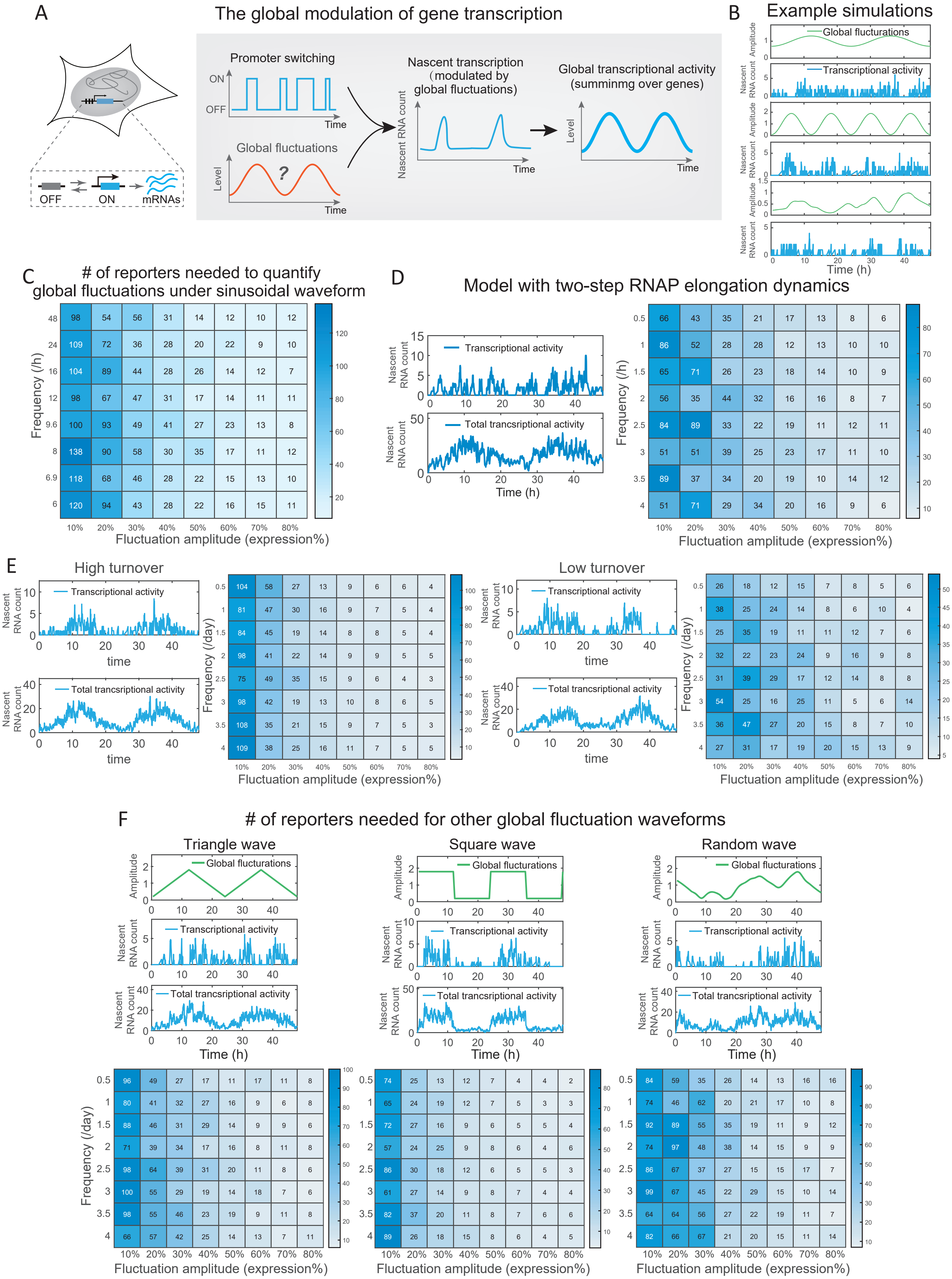
A
The global modulation of gene transcription
OFF
ON
mRNAs
Promoter switching
Time
Global fluctuations
Level
?
Nascent transcription (modulated by global fluctuations)
Nascent RNA count
Global transcriptional activity (summinmg over genes)
B
Example simulations
Global fluctuations
Transcriptional activity
Amplitude
Nascent RNA count
Time (h)
C
# of reporters needed to quantify global fluctuations under sinusoidal waveform
Frequency (/h)
Fluctuation amplitude (expression%)
D
Model with two-step RNAP elongation dynamics
Transcriptional activity
Total trancsriptional activity
Time (h)
Frequency (/h)
Fluctuation amplitude (expression%)
E
High turnover
Low turnover
time
Frequency (/day)
Fluctuation amplitude (expression%)
F
# of reporters needed for other global fluctuation waveforms
Triangle wave
Square wave
Random wave
Global flucturations
Transcriptional activity
Total trancsriptional activity
Time (h)
Frequency (/day)
Fluctuation amplitude (expression%)

Figure S2 | Simulation-assisted design of the multi-integrated reporter system.

(A) Cartoons illustrating the conceptual design principle of the reporter system. A two-state model is often used to describe switches between promoter OFF and ON states (left). Gene-specific fluctuations affect individual genes, while gene-nonspecific global fluctuations can affect the transcription rate globally across genes, leading to temporally coordinated transcriptional activity dynamics across genes (middle). Summing over a sufficient number of genes would in theory remove gene-specific fluctuations, as they are uncorrelated between genes (right).

(B) Example simulated gene transcriptional activity dynamics under the modulation of different global fluctuations as indicated. From such a single-gene dynamics, it is almost implausible to extract the information on global fluctuations, as they convolute with gene-specific fluctuations to produce very noisy transcriptional dynamics.

(C) A heatmap showing the minimal number of reporter copies needed to extract global fluctuations with indicated characteristics. The minimal reporter copy number was determined by simulation (see **Materials and Methods**).

(D) Simulation results of a model whereby the mRNA synthesis reaction consists of two successive steps to account for the complexity in the RNAP elongation dynamics (see **Materials and Methods**).

(E) Simulation results of models whereby the promoter switching rates are altered. For the “High turnover” scenario, both k_on and k_off are five-fold of the original values, while for the “Low turnover”, both k_on and k_off are one-fifth of the original values.

(F) Simulation results for other global fluctuation waveforms. Both example simulated dynamics and the minimal reporter copy number are shown for each waveform. Note that the same model was used as in (B) and (C).

Figure S3

A List of reporter integration sites

position	gene
chr1: 23,059,275 - 23,059,361	KDMA1
chr1: 44,617,883 - 44,618,277	RNF220
chr3: 134,105,846 - 134,105,996	RYK
chr3: 136,235,326 - 136,235,596	
chr5: 59,875,492 - 59,875,788	PDE4D
chr7: 37,345,006 - 37,345,261	ELMO1
chr10: 42,598,050 - 42,599,651	ZNF33B
chr13: 72,929,210 - 72,929,250	PIBF1
chr20: 52,966,984 - 52,967,144	

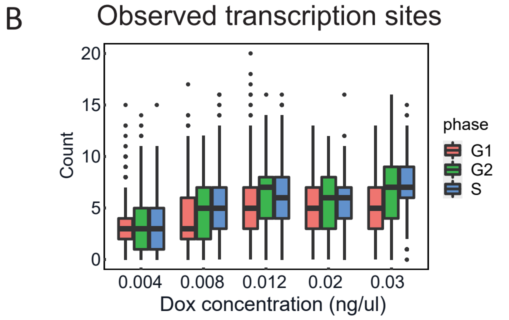

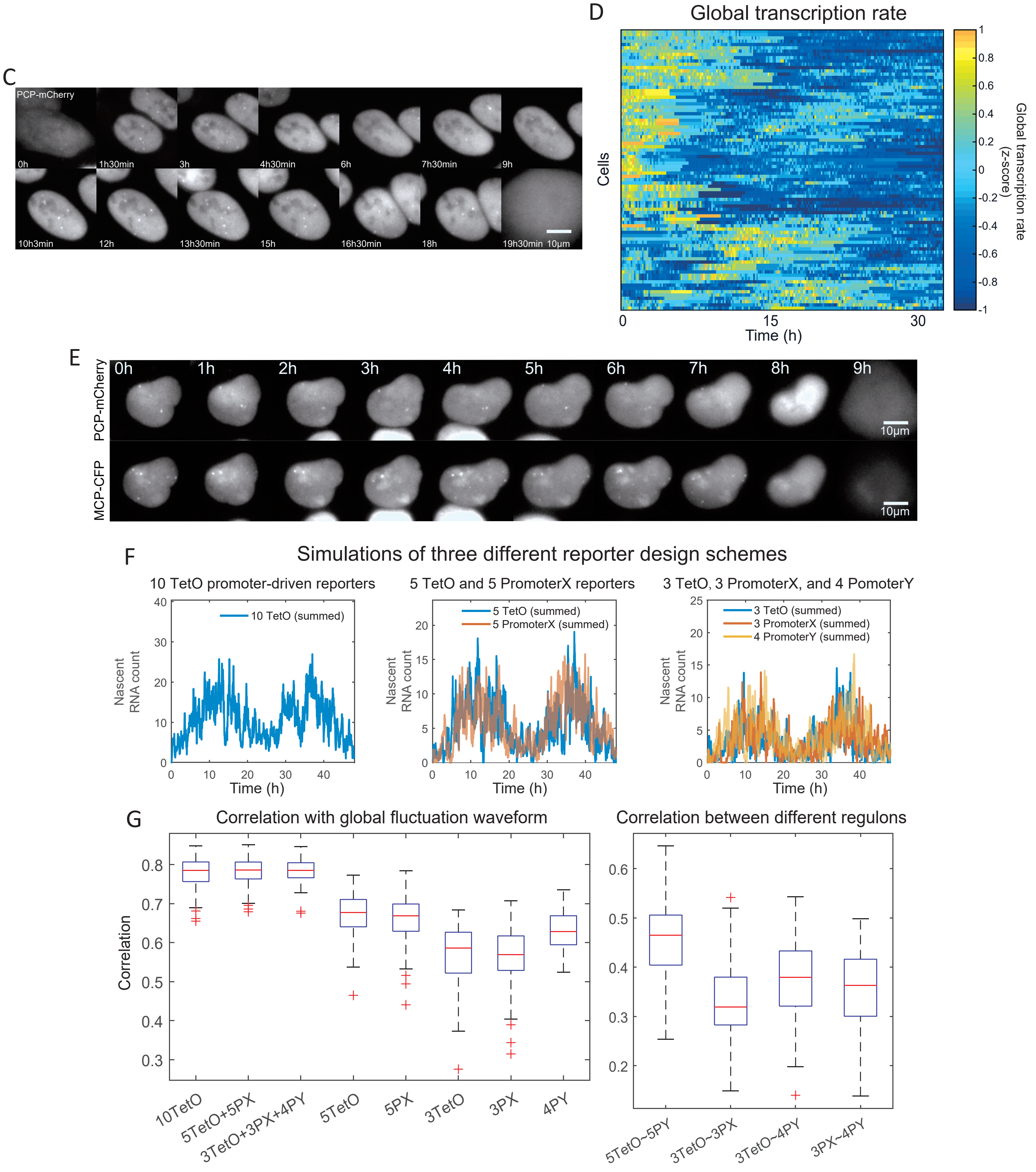

Figure S3 | Additional characterizations of the multi-integrated reporter system.

(A-B) Characterizations of identified and observed reporter sites. List of nine identified reporter integration sites using whole-genome sequencing of the reporter cell line **(A)**. Note that it is likely that not all integration sites were identified by sequencing, as we can observe more than nine sites during time-lapse imaging (**B**, n = 299 to 2862 cells in each bin**)**. Additionally, because reporter genes would duplicate during the S phase, we measured the copy number over the cell cycle and found that not all reporter duplicates at G2/M phase could be resolved by our imaging method. This result suggests that using summed transcriptional activity across detected gene loci, rather than the number of detected sites, is more appropriate for quantifying global transcription rate.

(C) Snapshots showing the example cell in **Figure 2B** at indicated time points.

(D) Heatmap showing z-scaled global transcription rate dynamics in individual cells (n = 85 cells under 0.012 ng/μL doxycycline).

(E) Two-color snapshots showing an example cell from **Figure 2D** at indicated time points (TetO/ACTB cell line).

(F) Summed transcriptional activity dynamics over regulons for three different reporter designs. For the first design, all promoter reporters are from the same regulon (TetO). And for the second and third designs, promoter reporters are from two or three separate regulons. In all simulations, the magnitude of sinusoidal global fluctuations is 70% of the reporter activity level. See **Materials and Methods** for details.

(G) Boxplots showing correlations of summed transcriptional activity dynamics from (F) with the input global fluctuation waveform for each simulated cell as well as correlations between different regulons within each simulated cell. n = 100 cells for each condition.

Figure S4

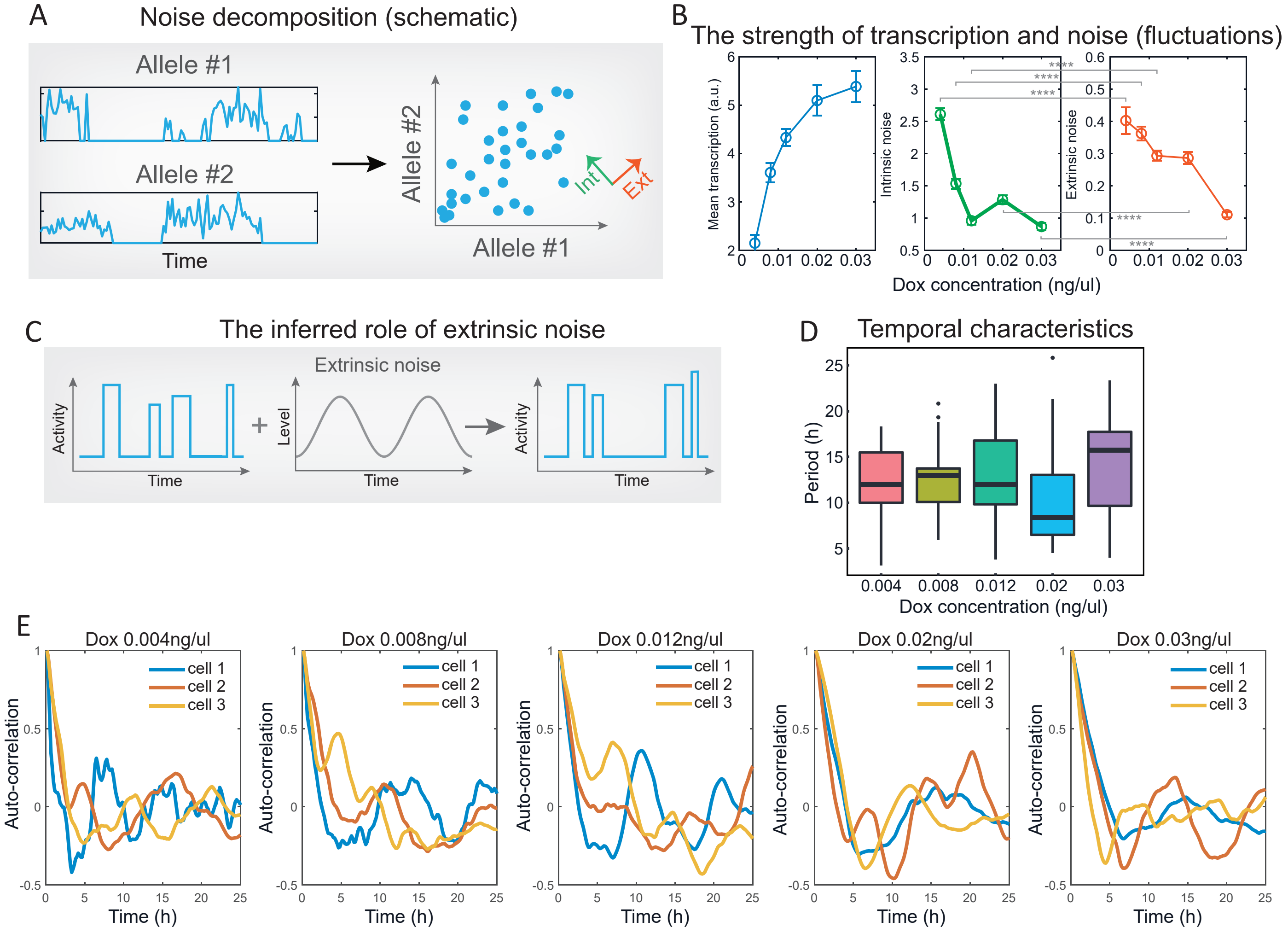

Figure S4 | Noise decomposition and temporal characterization of the multi-integrated reporter system.

(A-C) Decomposition of measured fluctuations into gene-specific (intrinsic) and gene-non-specific (extrinsic or global) components. Cartoons illustrating the conceptual schematic of the decomposition procedure **(A)**. Plots showing the strength of transcription and the strength of the associated fluctuations under indicated doxycycline conditions **(B)**. **** indicates $p<0.0001$ (paired-sample t-test). Based on results in **(B)**, the major role of extrinsic noise appears to be in the temporal organization of transcriptional bursts **(C)**.

(D) Boxplots showing periods in the global transcription rate dynamics under indicated doxycycline conditions (n = 19, 27, 42, 16, 16 cells from left to right). Period in each cell was estimated using the autocorrelation function of the rate dynamics (see **Materials and Methods**).

(E) Example auto-correlation functions of the single-cell global transcription rate dynamics for conditions indicated in **(D)**.

Figure S5

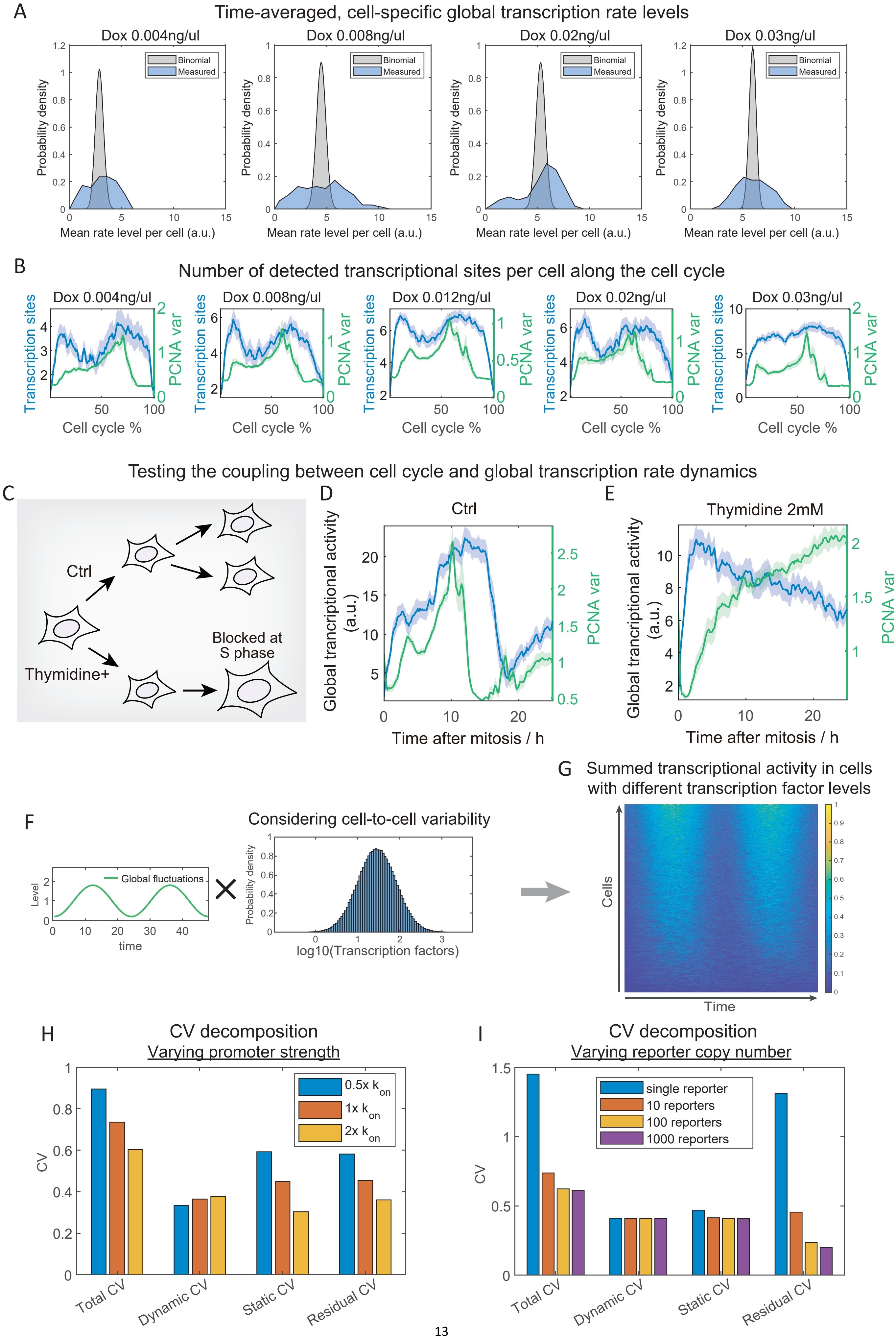
A
Time-averaged, cell-specific global transcription rate levels
Dox 0.004ng/ul
Dox 0.008ng/ul
Dox 0.02ng/ul
Dox 0.03ng/ul
Binomial
Measured
Probability density
Mean rate level per cell (a.u.)
B
Number of detected transcriptional sites per cell along the cell cycle
Dox 0.004ng/ul
Dox 0.008ng/ul
Dox 0.012ng/ul
Dox 0.02ng/ul
Dox 0.03ng/ul
Transcription sites
PCNA var
Cell cycle %
Testing the coupling between cell cycle and global transcription rate dynamics
C
Ctrl
Thymidine+
Blocked at S phase
D
Ctrl
Global trancriptional activity (a.u.)
PCNA var
Time after mitosis / h
E
Thymidine 2mM
Global trancriptional activity (a.u.)
PCNA var
Time after mitosis / h
F
Considering cell-to-cell variability
Global fluctuations
Level
time
Probability density
log10(Transcription factors)
G
Summed transcriptional activity in cells with different transcription factor levels
Cells
Time
H
CV decomposition
Varying promoter strength
0.5x k on
1x k on
2x k on
CV
Total CV
Dynamic CV
Static CV
Residual CV
I
CV decomposition
Varying reporter copy number
single reporter
10 reporters
100 reporters
1000 reporters
CV
Total CV
Dynamic CV
Static CV
Residual CV

Figure S5 | Additional characterizations of non-genetic heterogeneity in the global transcription rate.
(A) Distributions of time-averaged rate levels in individual cells under indicated conditions (n = 20, 20, 30, 26 cells from left to right) were compared to corresponding control distributions (analogous to **Figure 3C**, see **Materials and Methods**).
(B) Quantifications of the number of detected nascent transcriptional sites per cell during the cell cycle progression under indicated conditions. n = 20, 20, 51, 30, 26 cells from left to right and shading indicates ± SEM.
(C-E) Cell cycle blocking assay for testing the coupling between cell cycle and global transcription rate dynamics. Two populations of cells were either treated with thymidine (2mM) to block cells at S phase or untreated during time-lapse imaging **(C)**, and the resulting global transcription rate dynamics were aligned based on the first mitosis time after imaging in order to synchronize cell cycle phase in silico. Without cell cycle blocking, cells proceeded through normal cell cycles **(D**, n = 20 cells**)**, and in the presence of thymidine, cells were blocked at S phase as the PCNA signal remained at a high level **(E**, n = 41 cells**)**. Shading indicates ± SEM.
(F-I) Using simulations to recapitulate the deconvolution of heterogeneity in the estimated global transcription rate. In the previous simulations, we did not consider cell-to-cell variability in the global transcriptional activity arising from variations in cellular components (such as transcription factors). Here, we considered such variability and assumed that each cell has a pre-set transcription factor level **(F)**. Thus, the summed transcriptional activity dynamics are cell state-dependent **(G)**. Applying the deconvolution method as in **Figure 3H** allowed us to decompose different components of heterogeneity in the global transcription rate dynamics under scenarios when we altered the promoter strength **(H)** or the copy number of reporters **(I)**.

Figure S6

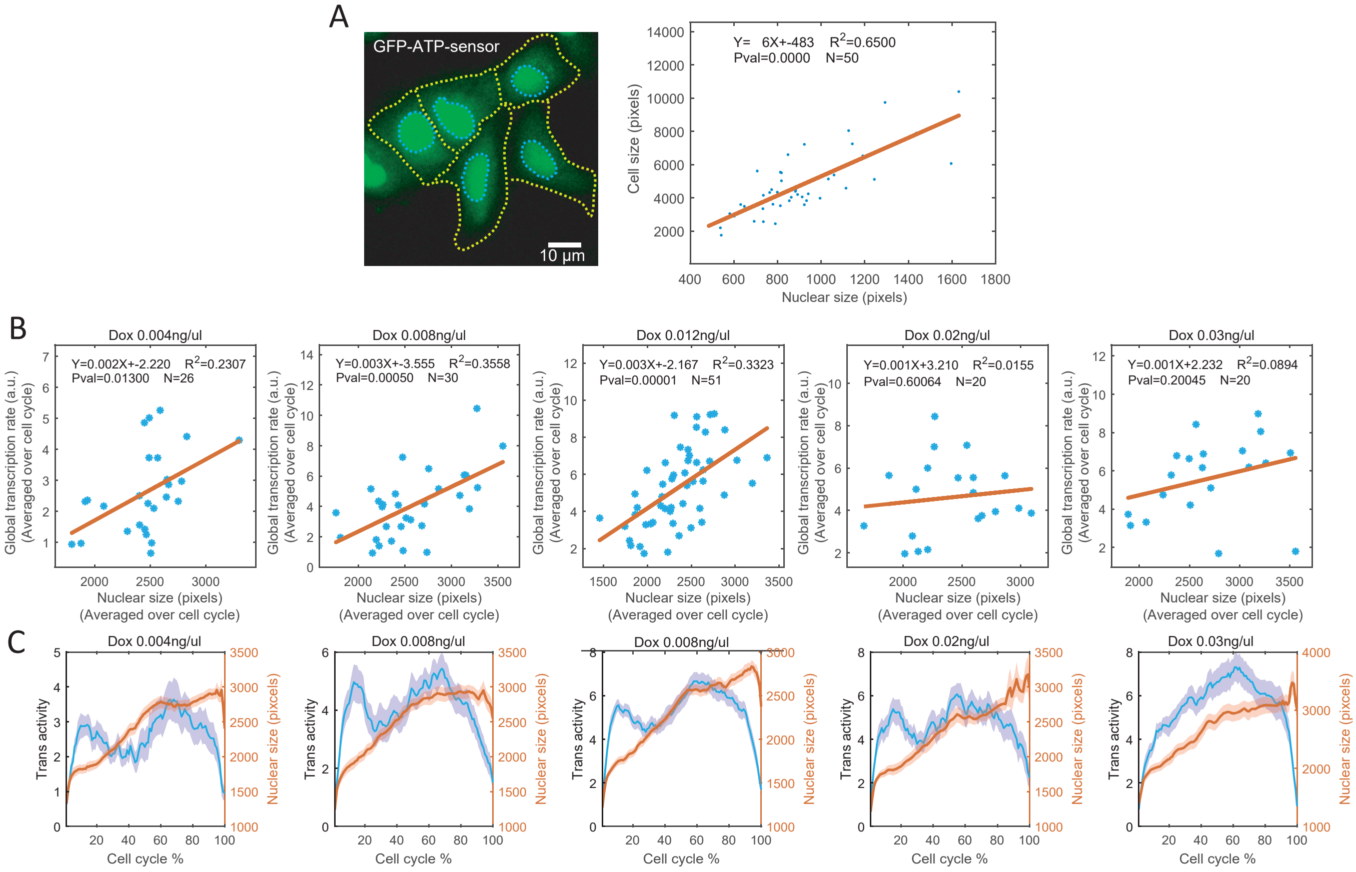

Figure S6 | The relationship between the estimated global transcription rate and nuclear size.

(A) The correlation between nuclear size and cell size. Fluorescence images of the ATP sensor cell line were used to determine both the cell size (i.e., the area within the yellow boundary) and nuclear size (i.e., the area within the blue boundary). n = 50 cells.

(B) Scatter plots showing the relationship between cell-cycle-averaged nuclear size and global transcription rate in individual cells. Red lines are linear fits.

(C) Population-averaged nuclear size dynamics and global transcription rate dynamics over the cell cycle under indicated conditions. Shading indicates ± SEM.

Figure S7

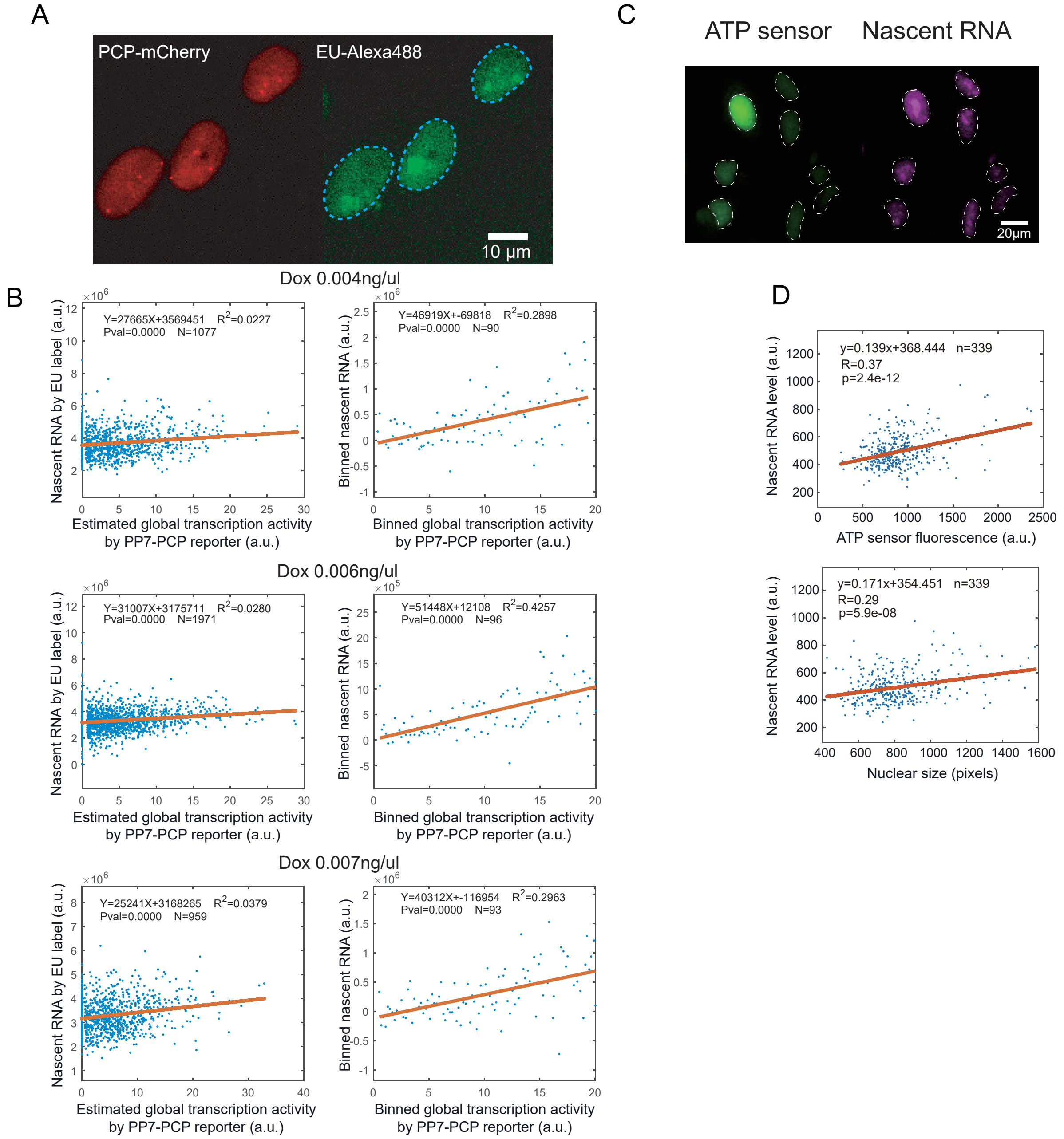

Figure S7 | Nascent RNA quantification by metabolic labeling.

(A-B) Nascent RNA quantification by metabolic labeling in the transcriptional reporter cell line. Cells were incubated with 5-EU (5-Ethynyl Uridine), followed by click-labeling using Alexa488 dye. PCP-mCherry foci signals allowed estimating the global transcriptional activity while the Alexa488 signals allowed quantifying the nascent RNA level **(A)**. See **Materials and Methods**. Scatter plots **(B)** showing the correlations between nascent RNA signal levels and the estimated global transcriptional activity (left) or the correlations between the two measures when cells were binned evenly based on the global transcriptional activity levels (right). The numbers of cells were as indicated for each doxycycline condition.

(C-D) Quantification of the relationship between ATP biosensor intensity and nascent RNA intensity in single cells. Two-color snapshots showing individual U2OS cells transfected with ATP biosensor (NLS-iATPSnFR1.0) and pulse-labeled with 5-Ethynyl Uridine (EU), followed by click chemistry-based labeling of EU **(C)**. Scatter plots showing ATP biosensor intensity (or nuclear size) and nascent RNA intensity in the same single cells (**D**, n =339 cells).

Figure S8

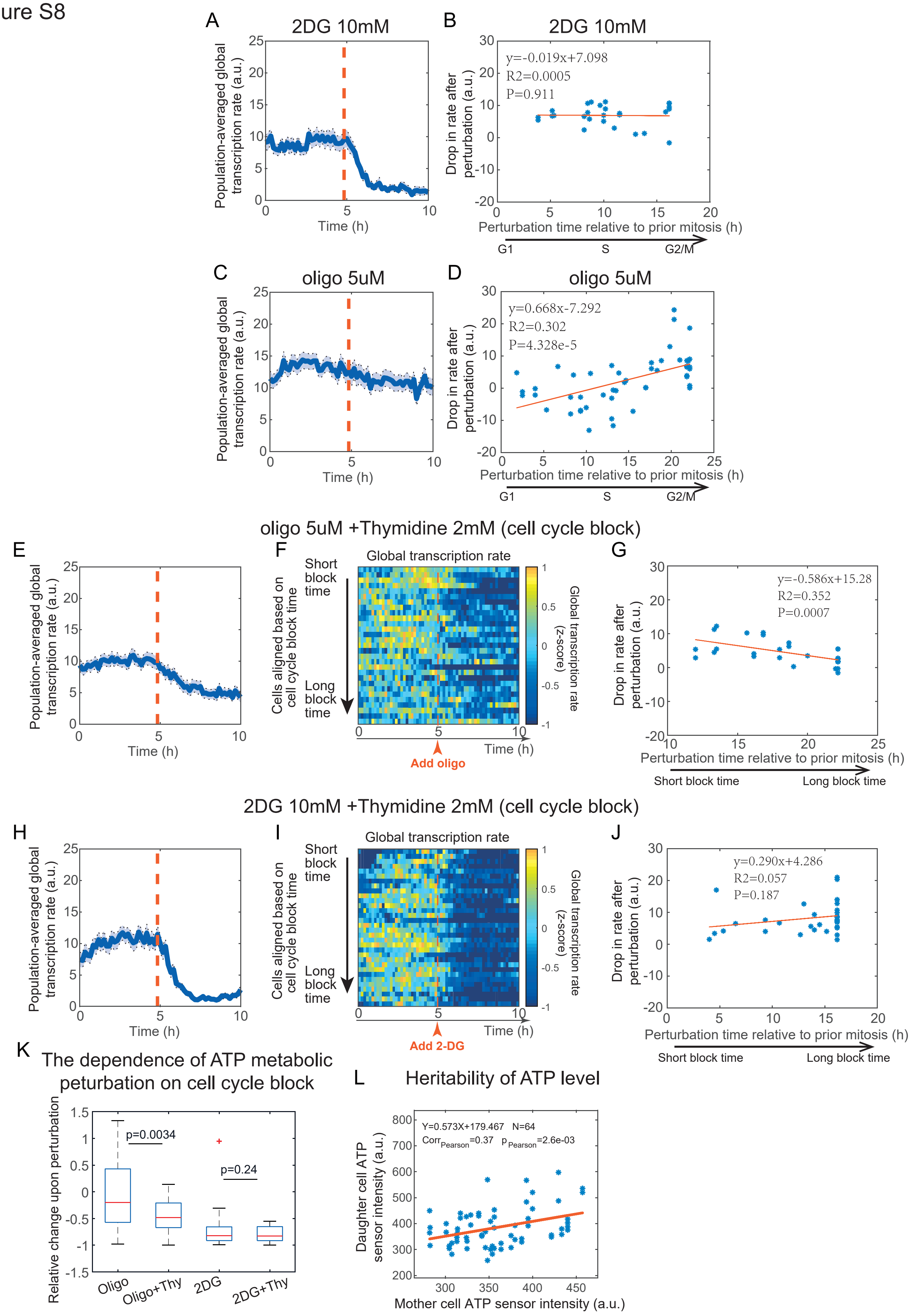

A
2DG 10mM
Population-averaged global transcription rate (a.u.)
Time (h)
B
2DG 10mM
y=-0.019x+7.098
R2=0.0005
P=0.911
Drop in rate after perturbation (a.u.)
Perturbation time relative to prior mitosis (h)
G1
S
G2/M
C
oligo 5uM
Population-averaged global transcription rate (a.u.)
Time (h)
D
oligo 5uM
y=0.668x-7.292
R2=0.302
P=4.328e-5
Drop in rate after perturbation (a.u.)
Perturbation time relative to prior mitosis (h)
G1
S
G2/M
oligo 5uM +Thymidine 2mM (cell cycle block)
E
Population-averaged global transcription rate (a.u.)
Time (h)
F
Global transcription rate
Cells aligned based on cell cycle block time
Short block time
Long block time
Global transcription rate (z-score)
Time (h)
Add oligo
G
y=-0.586x+15.28
R2=0.352
P=0.0007
Drop in rate after perturbation (a.u.)
Perturbation time relative to prior mitosis (h)
Short block time
Long block time
2DG 10mM +Thymidine 2mM (cell cycle block)
H
Population-averaged global transcription rate (a.u.)
Time (h)
I
Global transcription rate
Cells aligned based on cell cycle block time
Short block time
Long block time
Global transcription rate (z-score)
Time (h)
Add 2-DG
J
y=0.290x+4.286
R2=0.057
P=0.187
Drop in rate after perturbation (a.u.)
Perturbation time relative to prior mitosis (h)
Short block time
Long block time
K
The dependence of ATP metabolic peturbation on cell cycle block
Relative change upon perturbation
p=0.0034
p=0.24
Oligo
Oligo+Thy
2DG
2DG+Thy
L
Heritability of ATP level
Y=0.573X+179.467 N=64
Corr Pearson=0.37 p Pearson=2.6e-03
Daughter cell ATP sensor intensity (a.u.)
Mother cell ATP sensor intensity (a.u.)

Figure S8 | Additional evidence supporting the role of ATP metabolism.

(A-D) Additional characterizations of transient responses to perturbations in ATP metabolism. Population-averaged responses to the addition of glycolysis inhibitor, 2DG (**A**, n = 27 cells). Shading indicates ± SEM. Scatter plot showing the drop in global transcription rate in response to the drug perturbation, calculated by after-perturbation intensity minus before-perturbation intensity (**B**, n = 27 cells). Cells were horizontally aligned according to their cell cycle phases at the time of perturbation. Quantifications of responses to oxidative phosphorylation inhibitor oligomycin (**C-D,** n = 49, 49 cells), analogous to **A-B**.

(E-G) Characterizations of the effect of cell cycle block on the transient responses to the inhibition of oxidative phosphorylation. Population-averaged responses to the addition of oligomycin at 5-hour post imaging (**E**, n = 29 cells). Shading indicates ± SEM. Note that thymidine was added 24 hours prior to the start of the imaging to block the cell cycle. Heat map showing z-scaled global transcription rate dynamics in individual cells before and after the perturbation (n = 29 cells) **(F)**. Cells were vertically aligned according to their blocked time in S phase at the time of perturbation (i.e., time relative to prior mitosis). Scatter plot showing the drop in global transcription rate in response to the drug perturbation, calculated by after-perturbation intensity minus before-perturbation intensity (**G**, n = 29 cells). Cells were horizontally aligned according to the time relative to prior mitosis.

(H-J) Analogous characterizations to **E-G** for the inhibition of glycolysis. n = 32 cells.

(K) Boxplots summarizing results in **A-D** and **E-J**. Cell cycle inhibitor thymidine sensitized the response of global transcription rate to the inhibition of oxidative phosphorylation (by oligomycin), but not to the inhibition of glycolysis (by 2DG). P-value from Welch's *t*-test.

(L) Scatter plot showing the relationship between ATP biosensor intensities from pairs of mother and daughter cells (n = 64 pairs).

Figure S9

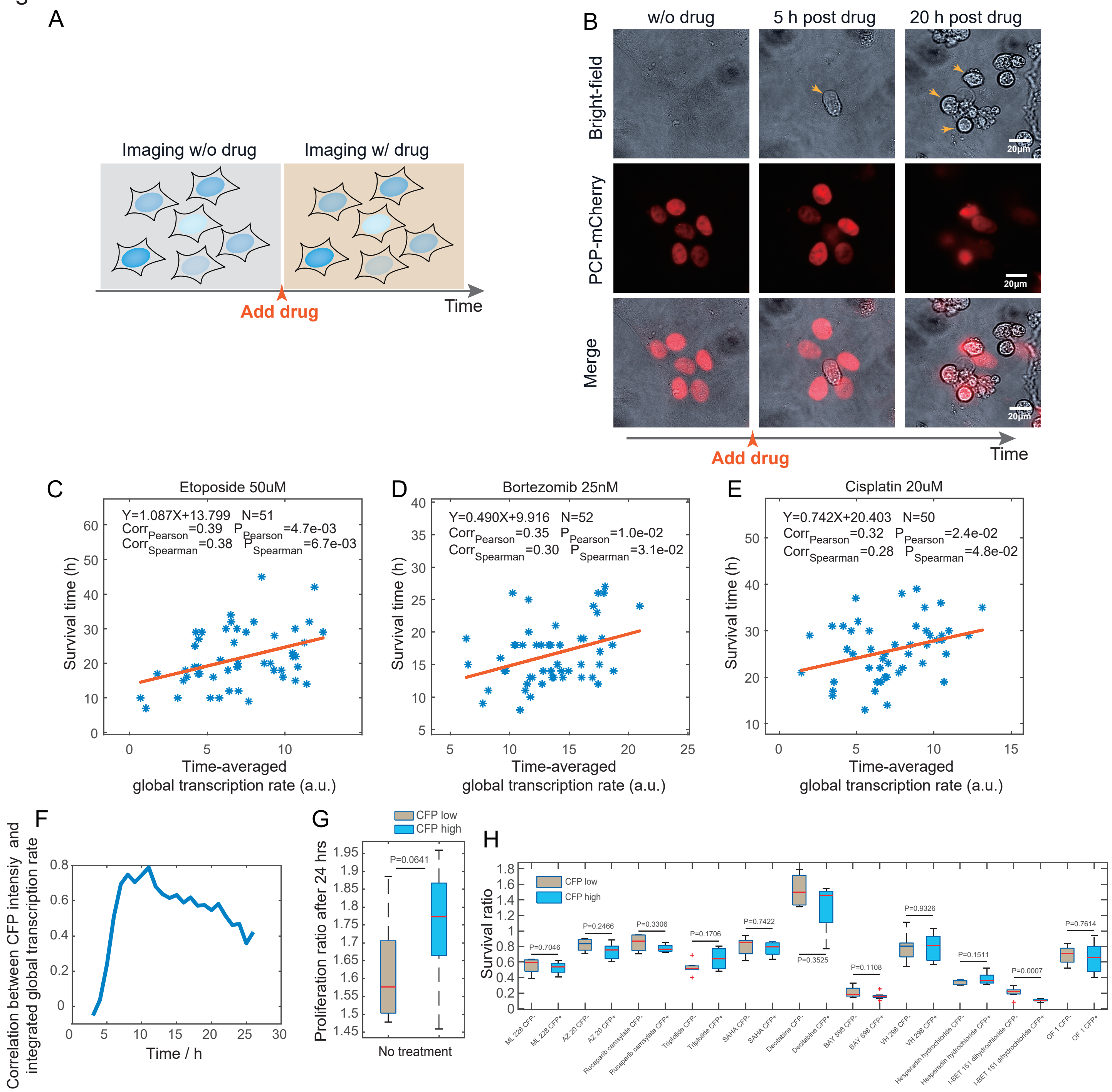

Figure S9 | The association of intra-clonal variability in global transcription rate with intrinsic drug tolerance.

(A) Cartoon illustrating the experimental scheme.

(B) Example snapshots showing cells imaged before and after drug perturbation (50 μM etoposide). Prior to drug addition, cells were imaged for at least one cell cycle for quantifying time-averaged rate levels. Yellow arrows indicated dying cells.

(C-E) Scatter plots showing the relationships between survival time in drug and time-averaged global transcription rate levels in individual cells under three different chemotherapeutic drugs, etoposide **(C)**, bortezomib **(D)**, and cisplatin **(E)**.

(F) Correlation between instantaneous CFP intensity and integrated global transcription rate. See Materials and Methods for details.

(G) Data from analogous experiments in Fig. 6B but without drug treatment. n = 10.

(H) Boxplots analogous to Fig. 6B for 11 additional drugs tested. n = 4-9. See Materials and Methods for details and Table S1 for information on concentrations.

Figure S10

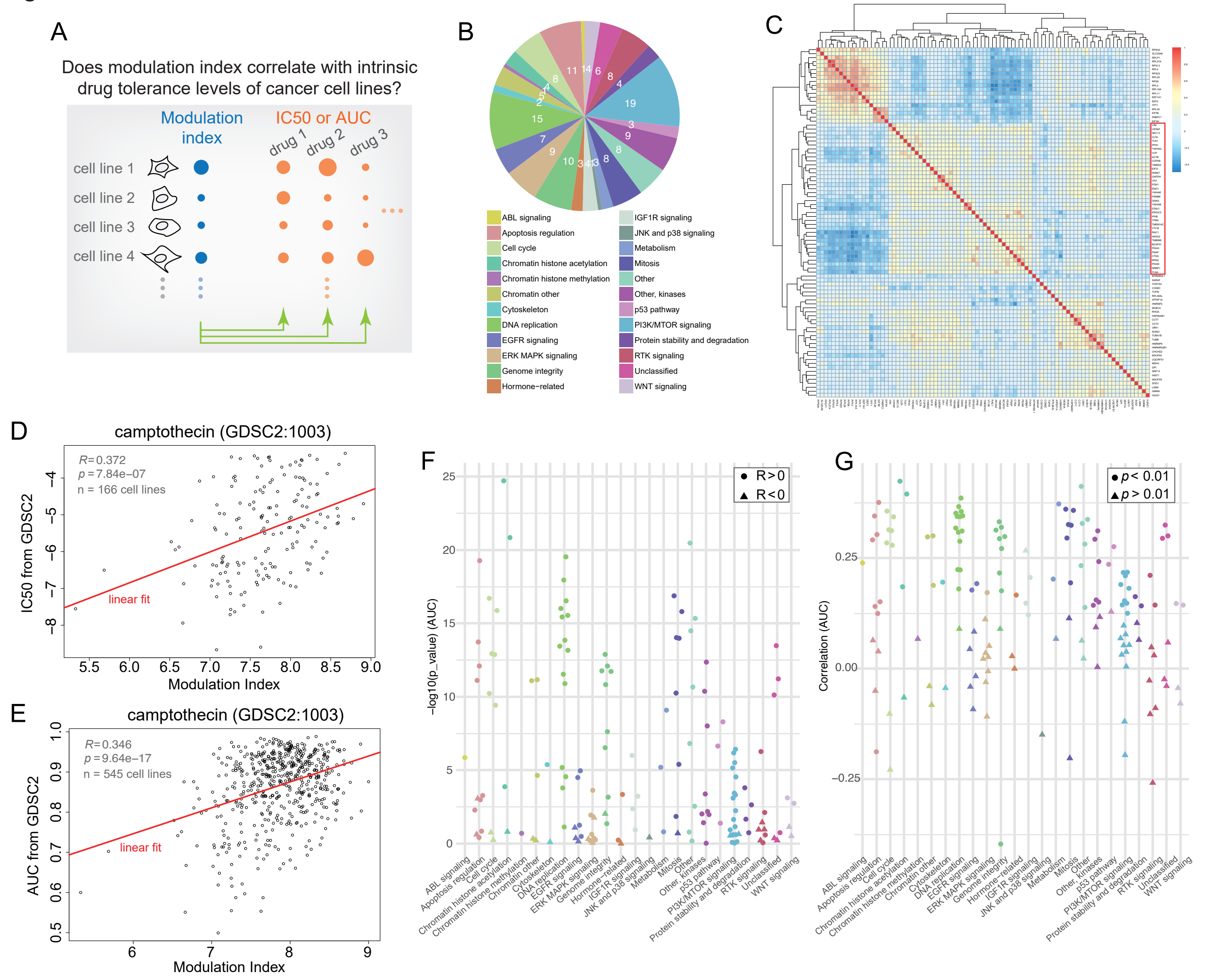

Figure S10 | Analyses of cancer cell line data implicated the role of global transcription rate modulation.

(A) Cartoons illustrating the main concept of the analysis. The analysis aimed to find out whether pre-existing differences in cancer cell lines' modulation indexes would correlate with the degrees of multi-drug tolerance.

(B) Pie chart summarizing drugs included in our analysis. Drugs were chosen based on the availability of both drug tolerance measures and target cell lines' RNA-seq data. A total of 153 drugs from 24 categories from the GDSC2 collection were included (**Materials and Methods**).

(C) Heatmap showing hierarchical clustering based on expression correlation of the top 100 genes from GEINE3 inference across 597 cancer cell lines. Red box indicates genes in the largest module (n = 38 genes), whose median expression level in each cell line was used as the "modulation index" (a proxy for the modulation of global transcription rate).

(D-E) Scatter plots showing the relationships between IC50 **(D)** or AUC **(E)** values and modulation index in individual cell lines.

(F) Dot plot showing the p-value from Pearson correlation calculated as in **(E)** for all included drugs. The same colormap as in **(B)** was used. The shape of the symbol indicates the sign of the Pearson correlation coefficient.

(G) Dot plot showing the Pearson correlation coefficient calculated as in **(E)** for all included drugs. The same colormap as in **(B)** was used. The shape of the symbol indicates the range of p-value.

Supplemental Table

Table S1 | List of anti-cancer drugs tested in our study. A total of 29 drugs from the Tocriscreen Epigenetics Library were tested. For each drug, the concentration and the estimated time for the drug to be effective in cell killing were provided (where null indicates no killing was observed during the time course).

Drug Name	Experiment concentration	Estimated lethal time (h)
Etoposide	50uM	24
Bortezomib	25nM	24
Cisplatin	50uM	24
A 485	50uM	null
Decitabine	50uM	72
Temozolomide	100uM	null
JQEZ5	2uM	null
ML 228	1uM	60
UNC 0642	2uM	null
Ryuvidine	10uM	null
BAY 598	100uM	24
OICR 9429	100uM	72
TP 064	10uM	null
VH 298	100uM	24
P 22077	2uM	null
AZ 20	10uM	60
Hesperadin hydrochloride	1uM	24
SD 1008	2uM	null
H 89 dihydrochloride	10uM	24
(+)-JQ1	10uM	null
I-BET 151 dihydrochloride	100uM	60
Bromosporine	10uM	60
OF 1	100uM	60
NVS-CECR2-1	1uM	null
Rucaparib camsylate	10uM	24
Triptolide	10uM	24
SAHA	10uM	24
JIB 04	2uM	null
Alexidine dihydrochloride	10uM	0 (immediately)

Supplemental Video

Video S1 | Time-lapse movie of the multi-integrated reporter system in an example cell. The cell was under 0.012 ng/μL doxycycline and the image of PCP was acquired at one frame per ten minutes. This cell was analyzed in Figure 2B. Available as a separate AVI file.
